# Systematic evaluation of PASEF acquisition strategies in complex metaproteomes

**DOI:** 10.1038/s41467-026-75845-5

**Published:** 2026-08-03

**Authors:** Feng Xian, Goran Mitulovic, Ranjith Kumar Ravi Kumar, Lukas Uhrik, Elisabeth Urbauer, Doriane Aguanno, Dirk Haller, Manuela Schmidt, David Gomez-Varela

**Affiliations:** 1 Center of Excellence for Metaproteomics University of Vienna - Bruker Daltonics, University of Vienna, Vienna, Austria; 2 Systems Biology of Pain, Division of Pharmacology & Toxicology, Department of Pharmaceutical Sciences, Faculty of Life Sciences, University of Vienna, Vienna, Austria; 3 Bruker Austria GmbH, Vienna, Austria; 4 Chair of Nutrition and Immunology, Technical University of Munich, Freising, Germany; 5 ZIEL - Institute for Food & Health, Technical University of Munich, Freising, Germany

**Keywords:** Proteomics, Microbiome

## Abstract

Metaproteomics measures functional expression in complex microbial communities, but extreme sample complexity and dynamic range challenge acquisition strategies. Trapped ion mobility spectrometry with parallel accumulation–serial fragmentation (PASEF) has expanded into multiple acquisition modes, yet systematic evaluations in high-complexity metaproteomes remain limited. Here, we benchmark five PASEF modes—DDA-, DIA-, Slice-, Synchro-, and midia-PASEF—using a complex fecal peptide background spiked with defined bacterial references. Across three gradients and input levels, 540 LC–MS acquisitions are analyzed under matched conditions. Based on data-derived performance scores, DIA-based strategies outperform DDA-PASEF in peptide and protein coverage, particularly for low-abundance microbial features. DIA- and Slice-PASEF show strong quantitative reproducibility, reduced ratio compression, and consistent species-abundance scaling, while functional profiling reveals expanded annotation depth. When tested in a murine colonic injury model, the two highest-scoring methods, DIA- and Slice-PASEF, capture concordant host and microbial responses.

## Abstract (web summary)

Metaproteomics uniquely reveals microbes’ and host cells’ function, therefore the ecosystem’s health. Decoding this complex dialogue requires optimal methods. Here, the authors benchmark five PASEF strategies and show which approaches best improve sensitivity, reproducibility, and functional insights.

## Introduction

Metaproteomics is increasingly used to investigate the functional interplay between complex microbial communities (e.g., gut microbiome) and their hosts^1,2^. Gut metaproteomics represents one of the most demanding application scenarios for LC–MS–based proteomics^3–5^. Fecal samples comprise dense mixtures of host and microbial proteomes spanning several orders of magnitude in abundance, providing a stringent stress test for acquisition strategies and data analysis pipelines^6^. At the same time, metaproteomic studies increasingly seek to quantify low-abundance community members and functional pathways that are central to host–microbiome interactions and diseases, placing exceptional demands on sensitivity, reproducibility, and quantitative accuracy^7–9^.

Trapped ion mobility spectrometry coupled with parallel accumulation–serial fragmentation (PASEF) has substantially expanded the speed, sensitivity, and quantitative precision of liquid chromatography–mass spectrometer (LC-MS)-based proteomics^10–16^. On this platform, multiple acquisition strategies have been developed, including data-dependent (DDA-PASEF^10^), data-independent (DIA-PASEF^11^), and more recent variants such as Slice-^12^, Synchro-^13^, and midia-PASEF^14^, which differ in their precursor sampling logic and trade-offs between analytical depth and throughput. However, the direct side-by-side comparison under matched analytical and computational conditions is missing. Moreover, despite their benefits in relatively low- to medium-complex samples, the performance of each acquisition strategy in highly complex biological matrices, where dynamic range, co-elution, and sampling bias strongly influence both identification and quantification^17^, is unknown.

PASEF technology applied to complex metaproteomes^18^ improves taxonomic and functional depth, increases throughput, improves limits of detection, and discovers host–microbiome interactions in preclinical mouse models of inflammatory bowel disease^1^. As a result, DDA- and DIA-PASEF are increasingly adopted in metaproteomic workflows to improve sequencing depth and throughput in gut microbiome studies^1,18–21^. Thus, it is necessary to study if the more recent methods^12–14^ (e.g., Slice-, Synchro-, midia-PASEF) could further provide improvements on identification depth, sensitivity to low-abundance species, quantitative reliability, and functional interpretation in complex microbiome samples^18,20^.

Here, we establish a controlled benchmarking framework to evaluate five PASEF acquisition strategies (PentaPASEF) across chromatographic regimes and sample input levels using a highly complex fecal peptide background spiked with defined reference bacteria. We systematically assess qualitative performance, quantitative precision and accuracy, species- and function-level consistency, and false-positive control across 540 matched LC–MS acquisitions. Finally, we evaluate the biological impact of acquisition choice in a murine epithelial injury model^22^, showing that DIA- and Slice-PASEF capture highly concordant microbial and host responses while differing primarily in quantitative sensitivity-related changes. Together, this study enables direct translation of acquisition design into expected analytical performance, providing practical guidance for benchmarking PASEF strategies according to sample complexity, sensitivity requirements, and experimental throughput.

## Results

### Experimental design for benchmarking PASEF acquisition strategies in fecal microbiomes

Ion mobility spectrometry adds an orthogonal dimension of separation to proteomics by resolving ions according to their collisional cross section, thereby improving selectivity, sensitivity, and quantitative precision. When combined with trapped ion mobility spectrometry (TIMS) and parallel accumulation–serial fragmentation (PASEF), this approach enables near-complete ion utilization and accelerated duty cycles, substantially enhancing proteome coverage. These advances have led to several PASEF acquisition strategies, including DDA-, DIA-, Slice-, Synchro-, and midia-PASEF, yet their relative performance in highly complex biological matrices remains insufficiently characterized.

**Fig. 1:**
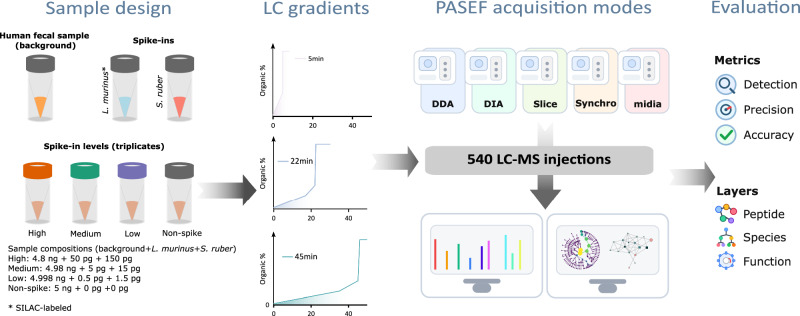
Benchmarking design for systematic evaluation of PASEF acquisition strategies. Human fecal peptides were used as a complex biological background and spiked with defined amounts of SILAC-labeled *Ligilactobacillus murinus* (*L. murinus*) and unlabeled *Salinibacter ruber* (*S. ruber*) at three input levels (50:150 pg, 5:15 pg, 0.5:1.5 pg) in triplicate. Samples were analyzed using five PASEF acquisition strategies (DDA, DIA, Slice, Synchro and midia) across three LC gradients (5, 22 and 45 min) in triplicate, together with non-spike controls.

To enable a systematic comparison, we established a controlled benchmarking framework spanning different chromatographic separations and sample complexities (Fig. 1). A pooled human fecal peptide sample was selected as the background matrix owing to its extreme proteomic complexity^4,7^, which captures the dynamic range and compositional heterogeneity typical of real biological samples. Two reference bacteria—SILAC-labeled *Ligilactobacillus murinus* (*L. murinus*) and unlabeled *Salinibacter ruber* (*S. ruber*)^1^—were spiked into the fecal peptide background at a fixed ratio (1:3) to emulate low-abundance community members. Three input levels (50 pg: 150 pg, 5 pg: 15 pg, and 0.5 pg: 1.5 pg) were prepared in triplicate and analyzed in triplicate to ensure statistical robustness, together with non-spiked controls.

LC–MS/MS data were acquired using three gradient lengths (5, 22, and 45 min) to balance throughput and depth across all PentaPASEF modes, resulting in 540 injections covering all experimental combinations. DDA-PASEF data were processed with MSFragger^23^ within FragPipe, whereas all other datasets were analyzed using DIA-NN^24^ to maximize comparability, applying a 1% FDR at precursor and protein group levels. To ensure confident identification of spiked bacterial peptides, any heavy-labeled *L. murinus* or *S. ruber* identifications observed in non-spike controls (gradient- and method-wise) were removed from the corresponding experimental runs, and background peptides falsely assigned with heavy labeling were filtered.

This experimental framework provides a rigorous and biologically relevant basis to compare PASEF acquisition strategies, capturing their relative performance across different chromatographic resolutions, acquisition modes, and analytical depths in metaproteomic contexts.

### Acquisition strategy shapes taxonomic and functional resolution in metaproteomics

**Fig. 2:**
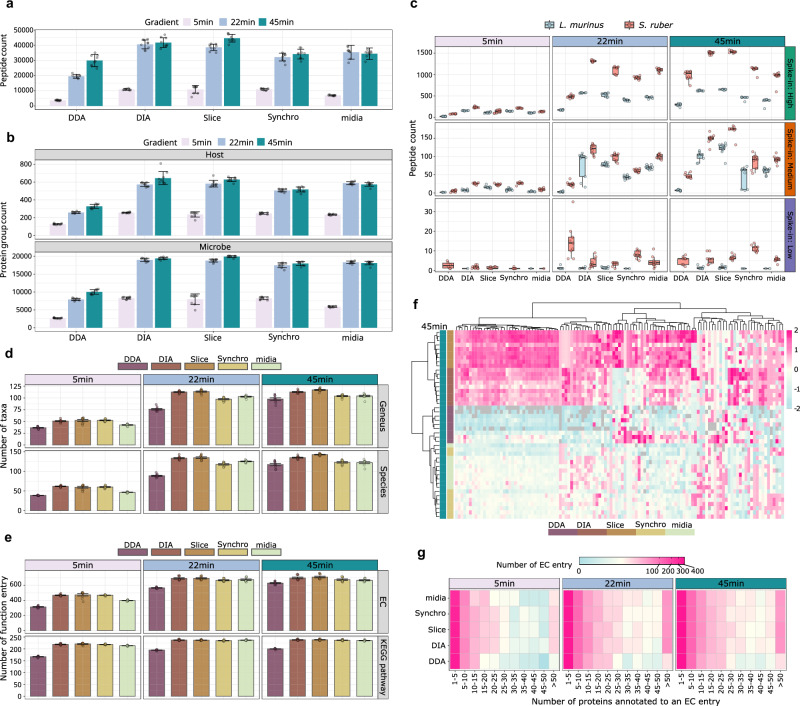
Qualitative performance of PentaPASEF across gradients and spike-in levels. Peptide (**a**) and protein group (**b**) identifications across acquisition methods and gradients. Bars represent mean identification numbers across spike-in conditions and spike-in replicates (*n* = 9, corresponding to three spike-in conditions with three spike-in replicates per condition), and the error bars show the standard deviations. **c** Identification of spiked *L. murinus* and *S. ruber* peptides across input levels. Box plots show the distribution of identified spike-in peptides across spike-in and acquisition replicates (*n* = 9 measurements per group, corresponding to three spike-in replicates measured with three acquisition replicates each). In each box plot, the centre line indicates the median; the box limits indicate the 25th and 75th percentiles; the whiskers extend to the smallest and largest values within 1.5 × the interquartile range. **d** Number of confidently annotated genera and species (≥3 taxon-specific peptides). The bars show the average number of genera and species across spike-in conditions and replicates (*n* = 9), and the error bars represent the standard deviation. **e** Number of enzymes (EC) and KEGG pathways detected. The bars show the average number of EC entries and KEGG pathways across spike-in conditions and replicates (*n* = 9), and the error bars represent the standard deviation. **f** Scaled peptide counts supporting taxonomic annotation for species shared across the five PASEF methods in spike-in conditions and replicates (*n* = 9 per method). Each cell represents the relative number of peptides assigned to a given species (in columns) within each method (in rows), with darker red indicating higher peptide support. Gray indicates species not detected in the corresponding replicates. **g** Distribution of microbial protein support per EC entry across acquisition methods and gradients. Protein support refers to the number of microbial proteins annotated to each EC entry, providing a measure of functional annotation depth. Source data are provided as a Source Data file.

We first assessed the qualitative performance of PentaPASEF acquisition strategies across LC gradients and spike-in levels. The overall number of identified peptides (Fig. 2a; Supplementary Data 1) and proteins (Fig. 2b) remained relatively stable across spike-in conditions and replicates (acquisition triplicates were averaged; *n* = 9 per bar), demonstrating the robustness of all PASEF methods. This stability was expected because the spiked bacterial peptides constituted only a minor fraction of the total peptide pool within the complex fecal background. Across PentaPASEF methods, peptide and protein identifications scaled consistently with LC gradient length, with 45-min separations yielding the highest coverage (Fig. 2a, b). DIA-based methods, particularly Slice- and DIA-PASEF, consistently exceeded DDA-PASEF in total identifications, reflecting their enhanced ion sampling efficiency and reduced precursor selection bias. Notably, even at the 5-min gradient, DIA-based modes maintained substantial peptide coverage, underscoring their suitability for high-throughput analyses. To further visualize global peptide-level differences among acquisition strategies, we performed PCA using peptide intensity profiles after averaging technical acquisition replicates within each gradient (Supplementary Fig. 1a). Samples clustered primarily by acquisition strategy, with DDA-PASEF separating most clearly from DIA-based methods and Slice-PASEF forming a distinct cluster among the DIA-derived approaches. This analysis indicates that PASEF modes differ not only in identification depth but also in the global peptide-level quantitative profiles they recover. For Slice- and midia-PASEF, alternative acquisition schemes were evaluated. A one-frame Slice configuration and a 5-Da overlay in midia-PASEF yielded higher identifications (Supplementary Fig. 1b) and were therefore retained for downstream analyses. Interestingly, midia-PASEF showed fewer identifications compared to DIA- and Slice-PASEF. To exclude analysis pipeline–dependent effects, DIA-PASEF and midia-PASEF datasets were reanalyzed with Spectronaut 20, which reproduced the same pattern (Supplementary Fig. 1c). Peptide-level overlap analysis revealed a large shared core among methods at all gradients, with overlap increasing at longer separations (Supplementary Fig. 1d–f). DDA-PASEF contributed the largest number of method-specific identifications, consistent with its precursor-selective acquisition strategy and broader precursor sampling space.

Focusing on the spiked *L. murinus* and *S. ruber* peptides, PentaPASEF methods displayed distinct sensitivity profiles (Fig. 2c; Supplementary Data 2). While following the gradient-dependent trend observed for total identifications, the number of spike-in peptide identifications decreased proportionally with input amounts. Nevertheless, DIA-based strategies generally retained higher recovery across gradients and spike-in levels, indicating improved detection of low-abundance peptides in complex matrices. Replicate variability increased at lower inputs (Fig. 2c), reflecting greater sampling fluctuation as signals approached the detection limit. Importantly, even at the sub-picogram level (0.5 pg *L. murinus*), DIA-based approaches—particularly DIA- and Slice-PASEF—still yielded detectable peptide identifications (Fig. 2c; Supplementary Data 2), most prominently at the 22- and 45-min gradients. Assessment of non-spike controls confirmed excellent false-positive control for both FragPipe and DIA-NN, with misassigned heavy-labeled *L. murinus* and *S. ruber* peptides below 1% across datasets (Supplementary Fig. 1g). DDA-PASEF showed slightly higher false-assignment frequencies, whereas DIA-based modes maintained tighter control.

Given that the background matrix consisted of human fecal material, we next examined how acquisition strategy influenced taxonomic and functional representation. Genus- and species-level profiles (Fig. 2d) and functional annotations based on EC numbers and KEGG pathways (Fig. 2e) mirrored the gradient-dependent trends observed for peptide identifications. The number of confidently assigned taxa (≥3 taxa-specific peptides) and functional entries increased with gradient length but differed only marginally between 22- and 45-min runs, indicating that near-maximal profiling depth was already achieved at 22 min. The recovered community composition was consistent with previous fecal metaproteome reports (Supplementary Fig. 1h).

Although DDA-PASEF detected a similar number of species as DIA-based methods (Fig. 2d), peptide-level inspection revealed that species shared across methods were supported by substantially fewer peptides in DDA datasets (Fig. 2f). This suggests lower annotation confidence and reduced taxonomic coverage for DDA-derived assignments, particularly compared with DIA- and Slice-PASEF. A comparable pattern was observed at the functional level: analysis of protein support per EC entry (Fig. 2g) showed that even at the 5-min gradient, DIA-, Slice- and Synchro-PASEF annotated many ECs with >20 proteins, whereas DDA- and midia-PASEF annotated far fewer. Moreover, DIA-based methods displayed a larger fraction of ECs supported by >50 proteins, indicating superior capacity to define enzymatic functions through multiple converging protein evidences. This broader protein-to-function mapping effectively enhances the functional detection limit in complex metaproteomic samples.

### Quantitative precision and accuracy across PentaPASEF strategies

**Fig. 3:**
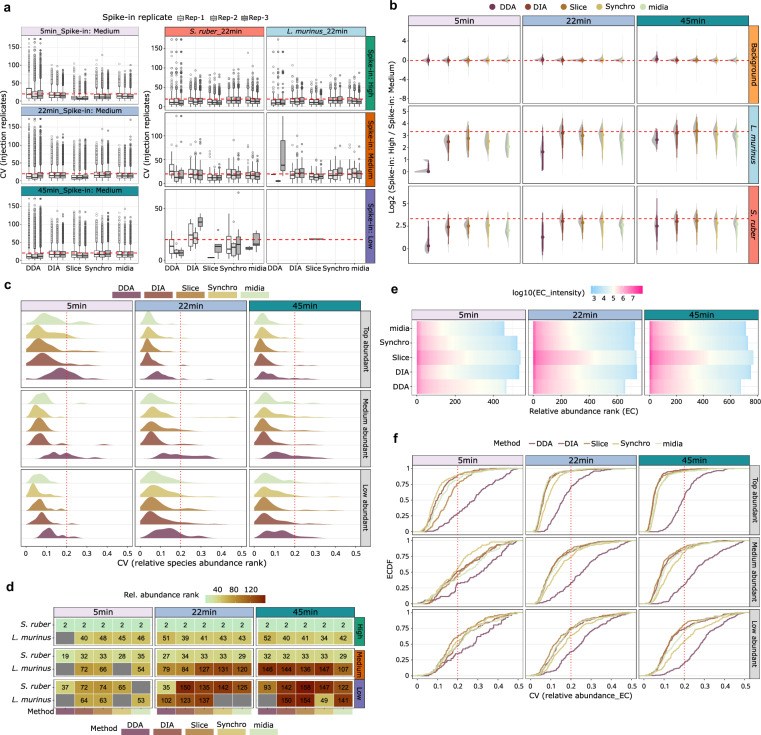
Quantitative performance of PentaPASEF strategies. **a** Distribution of peptide CVs (coefficient of variation) across acquisition replicates (*n* = 3 LC–MS acquisitions) for background and spike-in peptides. Box plots show the median as the centre line, the 25th and 75th percentiles as the lower and upper box limits, and whiskers extending to the smallest and largest values within 1.5 × the interquartile range. Data points beyond the whiskers are shown as individual points. Red dotted lines indicate a CV of 20%. **b** Distribution of peptide-level log₂ abundance ratios between high and medium spike-in conditions. Ratios were calculated from averaged peptide intensities across acquisition and spike-in replicates (*n* = 9 per group). The shaded density represents the distribution of peptide-level ratios for each method, gradient, and species group. Circles indicate the median, and the width of the vertical lines indicates the 70%, 80%, and 95% quantile intervals (from thicker to thinner). The red-dotted lines indicate the theoretical log₂ ratios. **c** Variability of species abundance ranks across replicates (*n* = 9) stratified into Top, Medium, and Low abundance tiers. The red-dotted lines indicate the CV of 20%. **d** Relative abundance ranks of spike-in species across gradients and input levels. **e** Dynamic range of EC entries across abundance ranks. The color bar represents the log_10_ intensity. **f** ECDF of CV distributions for quantified EC entries across abundance tiers (Top, Medium, and Low). The red-dotted lines indicate the CV of 20%. Source data for Fig. 3c–f are provided in the Source Data file.

Building on the qualitative benchmarks, we next compared the quantitative performance of the PASEF strategies under identical analytical conditions. Quantitative precision was assessed by calculating the coefficient of variation (CV) of peptide intensities across acquisition replicates (*n* = 3). Intra-group Pearson correlations were high for most datasets and gradients (Supplementary Fig. 2a). The few lower correlations observed for Slice-PASEF at the 22-min gradient (*r* < 0.9) were attributable to one high spike-in replicate, whereas lower correlations observed in the 45-min Slice- and midia-PASEF datasets were not linked to a consistent outlier pattern (Supplementary Data 3). Across all experiments, the majority of quantified peptides showed CVs below 20% (Fig. 3a), indicating overall high reproducibility of both the LC–MS platform and the acquisition schemes. Interestingly, DDA-PASEF displayed a pronounced gradient dependence, with variability decreasing at longer separations (average replicate-level median CV: 15.2% at 5 min, 11.5% at 22 min, and 9.64% at 45 min). In contrast, DIA-based methods showed less uniform gradient dependence; for example, Slice-PASEF maintained comparatively low median CVs at 5 and 22 min but showed higher variability at 45 min (average replicate-level median CV: 7.85%, 9.53% and 13.5%, respectively).

When analyzed separately for the spike-in species (Fig. 3a; Supplementary Fig. 2b), all methods showed good precision at the highest input (150 pg *S. ruber*, 50 pg *L. murinus*). At the medium level, performance declined first for DDA-PASEF, followed by Synchro- and midia-PASEF, particularly for 5 pg *L. murinus*. In contrast, Slice-PASEF largely preserved its precision under these conditions. At the lowest inputs (1.5 pg *S. ruber*, 0.5 pg *L. murinus*), variability increased markedly for all methods, indicating the current limits of reproducible quantification below the picogram range.

Quantitative accuracy was evaluated by comparing log₂ intensity ratios between high and medium spike-in conditions (Fig. 3b; Supplementary Data 4). For the human fecal background, the log₂ ratios were largely centered around zero across all methods and gradients, indicating consistent quantification for the majority of background peptides. Spike-in peptides showed clear separation consistent with the expected 10-fold difference. Under the 5-min gradient, all methods exhibited some ratio compression, most prominently for DDA-PASEF. For *L. murinus*, DIA-, Slice- and Synchro-PASEF clustered closely around the theoretical log₂ ratio of 3.3 at 22- and 45-min gradients, whereas DDA-PASEF showed broader dispersion and systematic underestimation. *S. ruber* peptides followed a similar pattern, with most DIA-based methods maintaining distributions closely centered near the expected ratio, while DDA-PASEF exhibited broader variability and stronger ratio compression. Among all methods, Slice-PASEF showed the narrowest ratio distributions and best agreement with theoretical expectations across gradients, indicating superior quantitative accuracy. Additional Spectronaut 20 analysis of representative DIA- and midia-PASEF datasets reproduced the main quantitative accuracy trends observed with DIA-NN for background and *S. ruber* peptides, while interpretation of *L. murinus* was limited by the lower number of quantified spike-in peptides (Supplementary Fig. 2c).

To assess quantitative consistency at the microbial compositional level, all quantified taxa were stratified into Top, Medium, and Low abundance tiers, and the CV of their relative abundance ranks across replicates (*n* = 9) was used as a measure of stability. Overall, DIA-based methods exhibited markedly tighter rank variability than DDA-PASEF across gradients and abundance tiers (Fig. 3c; Supplementary Data 5), consistent with the beta-diversity analysis, where DDA showed larger within-method dispersion (Supplementary Fig. 2d). Notably, the Top tier did not always display the lowest variability—particularly at 5 min—whereas several DIA-based modes maintained stable reproducibility even in Medium and Low tiers (Fig. 3c). We next examined the ranks of the spike-in species across conditions. As expected, both *S. ruber* and *L. murinus* showed a progressive decrease in rank with lower spike-in amounts, consistent with their relative spike-in levels (Fig. 3d). Overall, the rank patterns were largely comparable among most PASEF methods at each gradient, indicating similar quantitative scaling across acquisition strategies. However, ranking deviations were observed at low inputs, particularly for DDA-PASEF, which occasionally yielded disproportionately high ranks at low inputs—for example, ranking *S. ruber* at the 35th in the 22-min gradient, while other methods placed it behind the 120th. At the medium input, *L. murinus* ranked near 80th in DDA- and DIA-PASEF but around 130th in Slice-, Synchro- and midia-PASEF, and these differences largely diminished at the 45-min gradient, suggesting that longer separation times improve rank agreement among methods at moderate abundance levels (Fig. 3d). Across conditions, rank patterns were most consistent among DIA- and Slice-PASEF acquisitions, whereas DDA-PASEF frequently deviated at low input levels.

At the functional layer, the acquisition strategy strongly influenced quantitative depth. DIA-based methods, especially Slice-PASEF, exhibited a broader EC dynamic range and higher intensities at comparable abundance ranks (Fig. 3e; Supplementary Data 6), whereas DDA- and midia-PASEF showed weaker signals in low-abundance regions, suggesting reduced detection depth of enzymatic functions. This pattern was consistent across gradients, with minimal improvement beyond 22 min. When assessing functional quantification reproducibility, DIA-based methods again showed superior performance, characterized by steeper ECDF curves and a higher proportion of EC entries with CVs below 20%. Slice-PASEF consistently exhibited the most compact CV distributions across all abundance tiers (Fig. 3f), demonstrating its capacity to maintain precise functional quantification even for low-abundance ECs.

To translate the benchmark results into practical guidance, we summarized each acquisition strategy using data-derived normalized scores across key analytical dimensions, including identification depth, low-abundance sensitivity, quantitative precision and accuracy, taxonomic profiling, and functional annotation (Supplementary Fig. 3a, b). Scores were calculated separately for each gradient on a 1–5 scale, incorporating replicate variability as a penalty term. This overview confirmed DIA- and Slice-PASEF as the strongest overall performers, with DIA-PASEF offering the most balanced performance and Slice-PASEF providing particularly strong sensitivity and functional depth, whereas DDA-PASEF performed less favorably at shorter gradients, and Synchro- and midia-PASEF showed more context-dependent behavior (Supplementary Fig. 3a, b).

### DIA- and Slice-PASEF discover concordant microbial and host responses in a colon-injury mouse model

**Fig. 4:**
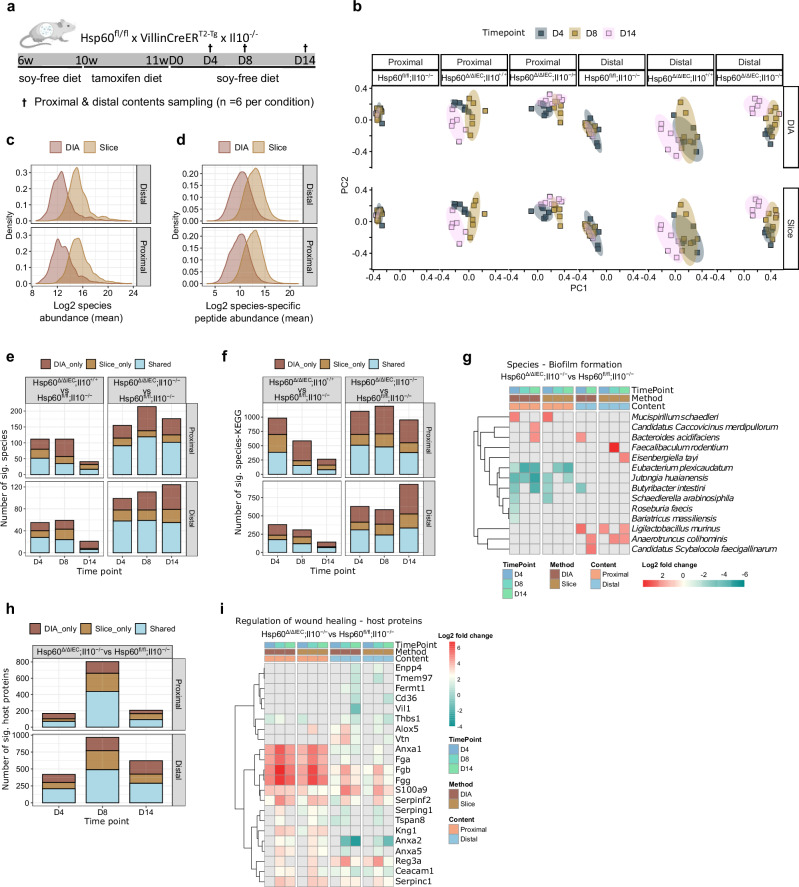
DIA- and Slice-PASEF reveal concordant microbial and host responses in vivo. **a** Schematic of the experimental design showing the strategy for the transient deletion of mitochondrial heat shock protein 60 (Hsp60) using tamoxifen induction in mouse intestinal epithelial cells (IECs) with/without interleukin 10 (Il-10) knockout. Proximal and distal contents were sampled and analyzed at day 4 (D4), day 8 (D8), and day 14 (D14) after the tamoxifen diet. Adapted from Xian F, et al^1^. under a CC BY 4.0 license. **b** Principal Coordinate Analysis based on Bray–Curtis dissimilarity of species-level relative abundance profiles quantified by DIA- and Slice-PASEF in proximal and distal colonic luminal samples across genotypes and time points. **c** Comparison of species abundance (mean) distributions between DIA- and Slice-PASEF. **d** Intensity distributions of species-specific peptides for DIA- and Slice-PASEF. **e** Number of significantly regulated species (adjusted *p* value ≤ 0.05) across time points and contrasts. Differential abundance was tested separately for each time point using limma linear models with empirical Bayes moderated two-sided t-tests. *P* values were adjusted for multiple comparisons using the Benjamini–Hochberg method. **f** Number of significantly regulated species–KEGG associations (adjusted *p* value ≤ 0.05) across time points and contrasts. Differential abundance was tested separately for each time point using limma linear models with empirical Bayes moderated two-sided t-tests. *p* values were adjusted for multiple comparisons using the Benjamini–Hochberg method. **g** Heatmap showing the log_2_ fold changes of species-KEGG associations for biofilm formation in the contrast of Hsp60^Δ/ΔIEC^;Il10^–/–^ versus Hsp60^fl/fl^;Il10^–/–^. Gray color in the plot represents the species-KEGG associations that were either statistically insignificant or missing. **h** Number of significantly regulated host proteins (adjusted *p* value ≤ 0.05) across time points in the contrast between Hsp60^Δ/ΔIEC^;Il10^–/–^ and Hsp60^fl/fl^;Il10^–/–^. Differential abundance was tested separately for each time point using limma linear models with empirical Bayes moderated two-sided t-tests. *P* values were adjusted for multiple comparisons using the Benjamini–Hochberg method. **i** Heatmap showing the log_2_ fold changes of host proteins involved in the regulation of wound healing. Gray color in the plot represents that the host proteins were either statistically insignificant or missing. Source data for Fig. 4b–i areprovided in the Source Data file.

DIA- and Slice-PASEF showed the best overall performance in the benchmarking experiment. We next investigated their ability to discover molecular mechanisms in an experimental design that integrates not only the complexity of a fecal microbiome but also the biological variability of an in vivo setup. Thus, we applied both acquisition strategies on a murine colon injury model created by the conditional knock-out of Hsp60 in intestinal epithelial cells combined with the presence or absence of Interleukin 10 (Il-10) expression (Fig. 4a). Conditional deletion of Hsp60 induces mitochondrial stress, leading to mucosal injury and microbial dysbiosis, while additional Il-10 deficiency impairs epithelial regeneration in the distal colon, resulting in persistent inflammatory pathology^1,22^.

Across all conditions, DIA- and Slice-PASEF identified a similar total number of peptides (Supplementary Fig. 4a; Supplementary Data 7) and the number of total bacterial species detected (Supplementary Fig. 4b; Supplementary Data 8). Alpha- and beta-diversity profiles (Supplementary Fig. 4c and Fig. 4b) show how both acquisition strategies discovered analogous injury-associated compositional gradients concomitant with the tissue phenotype separation between proximal and distal colon in Hsp60^Δ/ΔIEC^;Il10^–/–^ mice (e.g., different clustering only at D14 in the distal colon in Fig. 4b). While species-level abundances quantified by DIA- and Slice-PASEF were highly correlated (Pearson r > 0.91; Supplementary Fig. 4d), Slice-PASEF exhibited a systematic upward shift in species abundance distributions (Fig. 4c; median Slice–DIA abundance shifts of 2.51 and 2.48 log₂ units for distal and proximal samples, respectively). This shift was not driven by differences in the number of quantified species (Supplementary Fig. 4b) or species-specific peptides (Supplementary Fig. 4e). Instead, it reflected higher peptide-level intensities in Slice-PASEF (Fig. 4d; median Slice–DIA shifts of 2.52 and 2.59 log₂ units for distal and proximal samples, respectively), likely resulting from denser MS/MS sampling intrinsic to Slice acquisition and its propagation into MaxLFQ-based quantification. In fact, Slice-PASEF generated substantially larger raw file sizes and required longer DIA-NN processing times (Supplementary Fig. 4f). Minimum detectable species abundances were ~0.003% for both methods, with Slice-PASEF reaching slightly lower limits in both distal (0.0031% vs. 0.0034% in DIA) and proximal samples (0.0023% vs. 0.0037% in DIA) (Supplementary Data 9).

Differential abundance analysis revealed that the majority of significantly regulated species were shared between DIA- and Slice-PASEF across all comparisons and time points (Fig. 4e; Supplementary Data 10). Temporal patterns of differential species discovered the underpinnings correlating to the previously observed tissue pathology^22^ (Fig. 4e). In the Hsp60^Δ/ΔIEC^;Il10^+/+^ (injury at D4 and D8 but histopathological regeneration at D14) versus Hsp60^fl/fl^;Il10^–/–^ (no colitis at the time point in the absence of Hsp60 deletion^22^) contrast, the number of differential species markedly decreased by D14 in both colonic regions, consistent with epithelial regeneration due to the regain of Hsp60 protein expression in the conditional Hsp60^Δ/ΔIEC^;Il10^+/+^ mice at D14. In contrast, the comparison between Hsp60^Δ/ΔIEC^;Il10^–/–^ (injury at D4 and D8 without histopathological regeneration at D14 in the distal colon^22^) and Hsp60^fl/fl^;Il10^–/–^ (no colitis at the timepoint in the absence of Hsp60 deletion^22^) revealed a larger set of significant species at D14 in the distal colon compared to D4 and D8. These findings offer a new layer of information based on the ecosystem functional expression, which both increases the sensitivity, compared to imaging alternatives, to profile pathological changes and offers a plausible explanation for the recovery dynamics observed in these mice.

Interestingly, each method also detected a subset of unique significant species (Fig. 4e). Inspection of raw *p* values revealed that many method-unique species showed nominal differences in the alternative method but did not pass multiple-testing correction (Supplementary Fig. 4g; Supplementary Data 10). These features were therefore not statistically significant in the alternative method, suggesting that method uniqueness often reflected differences in statistical power or detection sensitivity rather than discordant biology. Direct comparison of Δ|log_2_FC| and Δ(−log_10_ *p*-value) further showed that method-unique calls aligned with the method exhibiting both larger effect sizes and stronger statistical evidence (Supplementary Fig. 4h), suggesting that uniqueness is primarily driven by method-dependent effect size estimation rather than increased measurement variability or statistical noise.

Despite partial species-level uniqueness, pathway-resolved analysis revealed strong functional concordance between DIA- and Slice-PASEF (Fig. 4f). Across contrasts and time points, both methods identified highly overlapping sets of significantly regulated species–KEGG associations, comprising 182 shared KEGG pathways linked to 175 shared species (Supplementary Data 11). In the Hsp60^Δ/ΔIEC^; Il10^–/–^ vs Hsp60^fl/fl^; Il10^–/–^ contrast, both methods consistently detected similar sets of inflammation-associated (e.g., NOD-like receptor signaling), stress-response metabolic (e.g., pentose phosphate and methane metabolism), and regeneration-associated pathways (e.g., biofilm formation and quorum sensing). A detailed analysis revealed how both acquisition strategies identify comparable species-level drivers underlying particular functional changes, as well as site-specific functional strategies of specific species. For example, in biofilm formation, DIA- and Slice-PASEF assigned highly similar sets of species concurrent with the lack of functional remodeling in the distal colon (Fig. 4g). Interestingly, both strategies discovered several species (e.g., *Eubacterium plexicaudatum*, *Butyribacter intestini*, and *Jutongia huaianensis*) in the proximal colon as associated with decreased biofilm activity, and other species (e.g., *Ligilactobacillus murinus* and *Anaerotruncus colihominis*) in the distal colon associated with increased biofilm formation. A similar concordance and site specification were also observed for the two-component system pathway (Supplementary Fig. 4i).

Analysis of the host proteome revealed interesting functional alteration patterns that were aligned with the above-described microbiome alterations. We observed highly concordant patterns of protein regulation between DIA- and Slice-PASEF (Supplementary Data 12), with both methods capturing maximal host protein dysregulation at D8, corresponding to the peak of tissue injury detected histopathologically as described previously^22^ (Fig. 4h, Hsp60^Δ/ΔIEC^;Il10^–/–^ vs Hsp60^fl/fl^;Il10^–/–^). Moreover, a larger number of proteins remained significantly altered at D14 in the distal compared with the proximal colon. Both methods consistently identified enrichment of epithelial repair and immune defense pathways, supporting concordant biological interpretation at the host level. Proteins involved in hemostasis (e.g., Fga, Fgb, and Fgg) and epithelial membrane repair and restitution (e.g., Anxa1, Anxa2, Anxa5) were more prominently upregulated in the proximal colon (Fig. 4i; Supplementary Fig. 4j), strongly suggesting accelerated tissue regeneration relative to the distal colon.

To connect microbial functional remodeling with the region-specific host response observed in the colonic injury model (Fig. 4h), we summarized significantly regulated microbial pathways from the Hsp60^Δ/ΔIEC^;Il10^–/–^ versus Hsp60^fl/fl^;Il10^–/–^ contrast at the KEGG BRITE category level (Supplementary Data 13). This analysis revealed sustained microbial functional remodeling across both colonic regions over time (Supplementary Fig. 5). Core metabolic categories, including amino acid, carbohydrate, energy, and lipid metabolism, remained altered from D4 to D14, indicating persistent changes in microbial metabolic activity during epithelial injury. Among host–microbe interaction-related categories, signal transduction showed a more consistently positive trajectory in the proximal colon than in the distal colon (Supplementary Fig. 5). This category includes bacterial two-component systems, which are widespread prokaryotic sensing modules that enable microbes to respond to environmental and host-derived cues and regulate stress responses^25^, nutrient acquisition, motility, biofilm formation, and virulence-associated behaviors. Thus, the proximal signal-transduction pattern may reflect sustained microbial environmental adaptation in a region where the host response shows stronger repair-associated features^22^ (Fig. 4h). Glycan biosynthesis and metabolism showed a distinct region- and time-dependent pattern, increasing early in the proximal colon but declining by D14, whereas the distal colon increased toward D14 (Supplementary Fig. 5). Mucin glycans provide binding sites and nutrient sources for mucus-associated bacteria and strongly shape host–microbiota interactions at the epithelial interface^26^. Therefore, this regional switch may reflect different mucus-interface states during injury and repair. In the proximal colon, the decline of glycan-related functions by D14 is consistent with reduced injury-associated mucus-interface remodeling, whereas the late distal increase may indicate continued remodeling of the mucus niche under persistent injury.

This interpretation is consistent with the host proteome analysis, which showed region-specific injury and repair programs at D14 (Fig. 4h, i; Supplementary Fig. 4j). In the distal colon, antimicrobial and inflammatory markers remained prominent, including increased Reg2/Reg3 (Supplementary Fig. 4j) and a stronger S100a9 response (Fig. 4i). In contrast, the proximal colon showed a more pronounced repair/resolution-associated signal, including strong upregulation of Anxa1 (Fig. 4i). Given the known roles of Reg-family proteins in epithelial antimicrobial defense^27^, S100a9 in neutrophil-associated intestinal inflammation^28^, and Annexin A1 in mucosal repair and inflammation resolution^29^, these patterns suggest that distal colon remains in a more inflammatory state, whereas proximal colon shows signs of repair-associated host adaptation.

## Discussion

The growing diversity of PASEF acquisition strategies has created both opportunity and uncertainty for metaproteomics^18,20,30^, as method choice increasingly determines analytical depth, quantitative reach, and biological interpretability. By benchmarking five PASEF implementations within a unified experimental and computational framework, our study reveals that acquisition strategy is not a neutral technical decision but a primary driver of metaproteomic sensitivity, reproducibility, and functional resolution. This study provides a comprehensive and biologically grounded evaluation of five PASEF acquisition strategies across chromatographic regimes, sample input levels, and analytical depths in fecal metaproteomes. By integrating qualitative, quantitative, and biological validation analyses, our results establish a unified framework for understanding how acquisition strategy selection influences identification depth, quantitative performance, and downstream biological interpretation in complex microbiome samples.

Across all acquisition modes, peptide and protein identifications scaled predictably with chromatographic separation length, confirming gradient duration as a primary determinant of analytical depth. DIA-based strategies consistently achieved broader and more stable peptide coverage than DDA-PASEF, including under high-throughput conditions (Fig. 2a, b). This advantage extended to the detection of low-abundance microbial peptides and functional annotations, where DIA-based acquisitions provided enhanced dynamic range and broader protein support per enzymatic entry (Fig. 2f, g). However, these gains come with practical trade-offs. Although Slice-PASEF achieved the strongest overall analytical performance in this study, this gain should be interpreted together with its practical costs. Compared with DIA-PASEF under identical processing settings, including search space, RAM, and CPU allocation, Slice-PASEF generated substantially larger raw files and required longer DIA-NN processing times (Supplementary Fig. 4f). These increases are consistent with the denser MS/MS sampling intrinsic to the Slice acquisition scheme. Thus, while Slice-PASEF provides clear advantages in sensitivity and quantitative depth, its deployment requires greater storage capacity, processing time, and data-management resources. These practical considerations are particularly relevant when scaling metaproteomic analyses to large cohorts or high-throughput study designs. In addition, midia-PASEF currently requires proprietary licensing, which may limit accessibility and adoption despite its demonstrated performance^14^. These considerations highlight that acquisition strategy selection should balance analytical performance against data management capacity and software availability.

Consistent with the data-derived guidance summary (Supplementary Fig. 3), the optimal PASEF strategy should be selected according to the primary experimental objective. For large-cohort metaproteomic studies, where throughput, reproducibility, and computational scalability are major priorities, DIA-PASEF provides a balanced option with high identification depth and robust quantitative performance. Slice-PASEF is particularly attractive when sensitivity and functional depth are prioritized, for example, in low-input or highly dilute samples such as environmental, marine, or host-associated microbiome samples with limited biomass. Although DDA-PASEF showed lower overall performance in the quantitative benchmark, it remains valuable for discovery-oriented workflows, microbial database construction, and de novo sequencing, where established analysis pipelines and cleaner precursor-resolved MS/MS spectra can support sequence interpretation. Synchro- and midia-PASEF showed more context-dependent performance in the present study, but remain conceptually promising. In particular, midia-PASEF offers high precursor specificity through mobility-encoded acquisition, and further optimization of window design, sample input, and software support may expand its utility for applications requiring confident precursor assignment, including database construction and de novo sequencing. Thus, the PASEF method of choice should be guided by whether the primary aim is cohort-scale throughput, maximal sensitivity, database generation, quantitative robustness, or method development.

The use of distinct computational ecosystems for DDA- and DIA-like acquisitions is an important consideration when interpreting benchmarking results. The main benchmark used established workflows available at the time of analysis, namely FragPipe/MSFragger for DDA-PASEF and DIA-NN for DIA-based methods. During manuscript development, DIA-NN introduced support for DDA data, allowing reciprocal cross-validation by reprocessing DDA-PASEF with DIA-NN and DIA-PASEF with the FragPipe/diaTracer workflow. These analyses showed that software choice affected absolute identification numbers, but the major acquisition-level trends were preserved (Supplementary Fig. 6a). Moreover, repeating the five-method peptide-overlap analysis with DDA-PASEF processed by DIA-NN confirmed that DDA-PASEF retained the largest method-specific peptide set (Supplementary Fig. 6b–d), indicating that the high number of DDA-specific identifications was not solely attributable to FragPipe/MSFragger processing.

Quantitative analyses revealed marked differences between acquisition modes. Slice-PASEF achieved the most favorable balance between sensitivity and quantitative robustness, yielding consistently low coefficients of variation, minimal ratio compression, and stable species-level abundance ordering across spike-in series (Fig. 3). DIA-PASEF also maintained robust quantitative performance but exhibited slightly higher variability under low-input conditions (Fig. 3a) and narrower dynamic ranges of quantified functions (Fig. 3e). In contrast, DDA-PASEF showed increased variability, especially under high-throughput conditions, ratio compression, and rank instability (Fig. 3b, c), underscoring the inherent limitations of precursor-dependent sampling in dense fecal peptide matrices. Our accuracy assessment was based on controlled spike-in ratios of two bacterial species and relative abundance rankings, which capture internal quantitative fidelity but do not provide absolute concentration ground truth. The two-species peptide spike-in was designed as a controlled benchmark rather than a microbiome-wide reference community. Establishing a universal microbiome-wide spike-in standard is challenging because microbial communities differ markedly across body sites, environments, and disease states, and because absolute detectability depends on organism-specific properties, including cell size, proteome size, lysis and extraction efficiency, digestion efficiency, peptide ionization, and database completeness. The use of two defined bacterial references, therefore, enabled controlled dilution, known ratios, and direct comparison of acquisition-dependent sensitivity and quantitative accuracy within a complex fecal peptide background. Accordingly, the spike-in results should be interpreted as relative performance benchmarks under controlled conditions, not as absolute sensitivity limits for all microbial taxa or microbiome types. This peptide-level benchmark complements our previous intact-cell spike-in study^1^, which assessed organism-level detectability in the presence of extraction and digestion effects.

Methodological differences observed in benchmarking also influenced the depth of biological interpretation. In a murine colonic epithelial injury model, DIA- and Slice-PASEF captured highly concordant microbial and host response patterns, which indicates that the acquisition strategy primarily modulates quantitative reach rather than biological directionality. More importantly, DIA- and Slice-PASEF datasets provided molecular evidence broadly consistent with the tissue pathology previously reported^22^ for the same samples. Nevertheless, the biological validation was conducted in a single disease model without an independent molecular ground truth for taxonomic change, pathway activation, or host response. Consequently, while relative concordance between methods can be assessed, the absolute biological correctness of specific response magnitudes cannot be definitively established, especially for method-dependent findings. Additional validation using orthogonal assays or controlled perturbation models will be required to fully define biological accuracy.

Although fecal samples represent one of the most compositionally complex and analytically demanding metaproteomic matrices, they do not capture the full diversity of microbiome-associated environments. Microbiomes from environmental^31,32^, oral^33,34^, or skin^35,36^ niches may differ in peptide complexity, dynamic range, and interference patterns, which could influence relative method performance. Spectral library size and sequence database design are important determinants of metaproteomic performance. In this study, we used a consistent sequence database and a predicted-library strategy across acquisition modes to minimize database- and library-dependent variation and focus on acquisition-dependent differences. However, different database sizes and spectral library designs may influence identification depth, quantitative behavior, and false-discovery control, and should be systematically evaluated in future metaproteomic benchmarking studies. Our benchmark was designed to compare practically deployable implementations of each PASEF strategy under matched metaproteomic conditions, rather than to exhaustively optimize the full acquisition-parameter space of every method. Accordingly, the results should be interpreted as a comparison of the tested implementations and not as absolute performance ceilings for each acquisition concept. For Slice-PASEF, the one-frame configuration was selected based on our preliminary evaluation and is consistent with the original Slice-PASEF report^12^, in which one-frame acquisition provided higher precursor and protein group identifications than two-frame acquisition under low-input conditions. By contrast, the relatively lower performance of midia-PASEF observed here should be interpreted as specific to the tested low-input fecal metaproteomic implementation. The original midia-PASEF^14^ benchmark was performed under substantially different conditions, including higher-input HeLa digest samples and a different instrument platform, and there is no published guidance for adapting midia-PASEF window schemes to low-input metaproteomic samples. Alternative window widths, overlaps, duty cycles, cycle designs, and dedicated processing strategies may therefore improve midia-PASEF performance in complex metaproteomic applications.

Our results indicate that DIA-based PASEF strategies—particularly DIA and Slice-PASEF—define a new operational regime for metaproteomics in which high-throughput workflows can simultaneously achieve deep coverage, stable quantification, and expanded functional detectability. Together, these findings establish a quantitative reference for selecting and refining PASEF strategies and provide a foundation for future development of acquisition designs, software tools, and standards that will enable reproducible, sensitive, and scalable metaproteomics.

## Methods

### Ethics

Human fecal sampling carried out at the University of Vienna was under the ethical approval (01149). Mouse work carried out at the Technical University of Munich, as well as the maintenance and breeding of mouse lines, was approved by the Committee on Animal Health Care and Use of the state of Upper Bavaria (Regierung von Oberbayern; AZ ROB-55.2-2532.Vet_02-14-217, AZ ROB-55.2-2532.Vet_02-20-58, and AZ ROB-55.2-2532.Vet_02-18-37) and performed in strict compliance with the EEC recommendations for the care and use of laboratory animals (European Communities Council Directive of November 24, 1986 (86/609/EEC)).

### Animals and housing conditions

Mice for in vivo experiments (Fig. 4) were male and housed under specific pathogen-free (SPF) conditions according to the criteria of the Federation for Laboratory Animal Science Associations (FELASA) (12-h light/dark cycles at 24–26 °C) in the mouse facility at the Technical University of Munich (School of Life Sciences Weihenstephan). All mice received a standard diet (autoclaved V1124-300, Ssniff) *ad libitum*, autoclaved water and were sacrificed by CO_2_ or isoflurane.

Details of the animal models can be found in our previous study^22^. Briefly, Hsp60^fl/fl^ mice and Hsp60^fl/fl^ x VillinCreER^T2-Tg^ mice were generated as described previously^37^ to create IEC-specific Hsp60 knockout mice via tamoxifen induction (Hsp60^Δ/ΔIEC^;Il10^+/+^). In addition, Hsp60^fl/fl^ and Hsp60^Δ/ΔIEC^;Il10^+/+^ mice were crossed with whole-body Il-10 knockout mice (Il10^-/-^) to generate Hsp60^fl/fl^;Il10^-/-^ and Hsp60^Δ/ΔIEC^;Il10^-/-^ mice^22^. For conditional Hsp60 deletion, mice and appropriate control mice (6-weeks of age) were kept on phytoestrogen-reduced diet 1005 (V1154-300, Ssniff) for four weeks under SPF conditions. Afterward, mice received 400 mg tamoxifen citrate per kg chow feed (CreActive T400 (10 mm, Rad), Genobios) *ad libitum* for 7 days. After the induction phase, the tamoxifen diet was replaced with the phytoestrogen-reduced diet. During and after the induction phase, mice were monitored daily and euthanized when a combined score considering weight loss, changes in stool consistency, general behavior, and the general state of health was reached. Animals were sacrificed at the indicated time points (D4, D8, and D14 after the tamoxifen diet). All mice and their respective genotypes were generated and maintained on an in-house crossing of C57Bl/6N and C57Bl/6J backgrounds.

### Protein extraction and SP3-assisted protein digestion for metaproteomics analysis

The procedures from protein extraction of gut microbiome material to final peptide preparation were performed as previously described^1,18^.

### Liquid chromatography-mass spectrometry configurations

Nanoflow reversed-phase liquid chromatography (Nano-RPLC) was performed on either ProElute or NanoElute2 systems (Bruker Daltonik, Bremen, Germany) coupled with timsTOF Ultra2 (Bruker Daltonik, Bremen, Germany) via the CaptiveSpray ion source, respectively. Mobile solvent A consisted of 100% water containing 0.1% FA, and mobile phase B consisted of 100% acetonitrile containing 0.1% FA.

### Liquid chromatogram setups

Results presented in Figs. 1–3 were generated with three LC gradients on the ProElute system. For 5-min gradient separation, peptides were loaded onto a PepSep® column (4 cm×150 µm) packed with 1.9 µm ReproSil C18 particles (Bruker). The mobile phase B was linearly increased from 2 to 35% in 5 min with a flow rate of 0.3 µL/min, followed by a steep increase to 95% in 0.1 minute. The mobile phase B was maintained at 95% for the last 3 min 0.3 µL/min. For 22-min gradient separation, peptides were loaded onto a PepSep® column (25 cm×75 µm) packed with 1.5 µm ReproSil C18 particles (Bruker). The mobile phase B was linearly increased from 2 to 20% in 17 min with a flow rate of 0.3 µL/min, followed by another linear increase to 35% within 5 min and a steep increase to 95% in 0.5 minute. The mobile phase B was maintained at 95% for the last 7.5 min 0.3 µL/min. For 45-min gradient separation, peptides were loaded onto a PepSep® column (25 cm×75 µm) packed with 1.5 µm ReproSil C18 particles (Bruker). The mobile phase B was linearly increased from 2 to 20% in 35 min with a flow rate of 0.3 µL/min, followed by another linear increase to 35% within 10 min and a steep increase to 95% in 1 min. The mobile phase B was maintained at 95% for the last 4 min 0.3 µL/min. Mouse colonic microbiome samples (Fig. 4) were analyzed on a NanoElute2 system coupled with an Aurora^TM^ ULTIMATE column (25 cm×75 µm) packed with 1.7 µm C18 particles (IonOpticks, Fitzroy, Australia) with a 45-min gradient. The mobile phase B was linearly increased from 2 to 20% in 35 min with a flow rate of 0.25 µL/min, followed by another linear increase to 35% within 10 min at a flow rate of 0.25 µL/min and then a steep increase to 95% in 0.5 min, with the flow rate increasing to 0.4 µL/min. The mobile phase B was maintained at 95% for the last 4.5 min at 0.4 µL/min.

### PentaPASEF method setups

Different PASEF methods can be extracted from the raw data uploaded. A summary report describing the general acquisition principle, precursor sampling strategy, and practical considerations for all five methods with key instrumental parameters is provided in Supplementary Data 14.

### Microbial database construction

To analyze the pooled human fecal samples, DDA-PASEF files generated in benchmarking were submitted to MSfragger^23^ (version 4.1) integrated in FragPipe computational platform (version 22.0), searching against the MGnify human gut protein catalogue (https://www.ebi.ac.uk/metagenomics/genome-catalogues/human-gut-v2-0-2)^38^. The decoy database was generated with reversed sequences. Trypsin was specified with a maximum of two missed cleavages allowed. The search included variable modifications of methionine oxidation and N-terminal acetylation and a fixed modification of carbamidomethyl on cysteine. The mass tolerances of 20 ppm were set for the precursor and fragment. Peptide length was set to 7 to 50 amino acids with a mass range from 500 to 5000 Da. The remaining parameters were kept as default settings. During the validation, MSBooster (version 1.2.31) was used for rescoring, and Percolator^39^ (version 3.6.5, default parameters) was used for PSM validation. FDR level was set to 1% for PSM, peptide, and protein. The identified proteins from the search formed a sample-specific protein database, containing 30854 protein sequences. For the benchmark comparison, this reduced microbial database was then combined with the reference proteomes of *Ligilactobacillus murinus* (UniProt UP000051612, accessed 2023-07-19), *Salinibacter ruber* (UP000008674, accessed 2023-07-19), *Homo sapiens* (UP000005640, accessed 2025-03-20), and contaminant sequences provided by DIA-NN, to generate the final combined FASTA search space. This combined FASTA file was used for DIA-NN predicted spectral library generation for DIA-, Slice-, Synchro-, and midia-PASEF analyses and for reprocessing the DDA-PASEF benchmark dataset with FragPipe/MSFragger. Thus, all benchmark acquisition methods were compared using the same underlying protein sequence search space.

To generate a sample-specific microbial database for the colonic samples (Fig. 4), we followed the recently published strategy of novoMP^1^. Briefly, a total of 60 µg pooled peptides (from both proximal and distal regions) were fractionated (Fisher Scientific, Cat. 84868) according to the manufacturer’s instructions. Eight peptide fractions were dried using vacuum centrifugation and then re-suspended in 30 µL of MS-grade water. The peptide concentration was measured in duplicate using a NanoPhotometer N60 (Implen, Munich, Germany) at 205 nm. Peptide samples were acidified with formic acid to a final concentration of 0.1% and were stored at -20 °C until LC-MS/MS analysis. 50 ng of each peptide fraction (a total of 8 fractions) were loaded on an Aurora^TM^ ULTIMATE column (25 cm×75 µm) packed with 1.6 µm C18 particles (IonOpticks, Fitzroy, Australia) with a total separation time of 60 min in DDA-PASEF mode. The TIMS analyzer was operated in a 100% duty cycle with equal accumulation and ramp times of 100 ms each. Specifically, 10 PASEF scans were set per acquisition cycle (cycle time of 1.17 s) with an ion mobility range from 0.7 to 1.3 (1/k0). The target intensity and intensity threshold were set to 20000 and 500, respectively. Dynamic exclusion was applied for 0.4 min. Ions with m/z between 100 and 1700 were recorded in the mass spectrum. Collision energies were dependent on ion mobility values, with a linear increase in collision energy from 1/K0 = 0.6 Vs/cm² at 20 eV to 1/K0 = 1.6 Vs/cm² at 63 eV. Fractionated DDA files were submitted to MSfragger^23^ (version 4.3) integrated in FragPipe computational platform (version 23.1), searching against the MGnify mouse gut protein catalogue (https://www.ebi.ac.uk/metagenomics/genome-catalogues/mouse-gut-v1-0). The decoy database was generated with reversed sequences. Trypsin was specified with a maximum of two missed cleavages allowed. The search included variable modifications of methionine oxidation and N-terminal acetylation and a fixed modification of carbamidomethyl on cysteine. The mass tolerances of 20 ppm were set for the precursor and fragment. Peptide length was set to 7 to 50 amino acids with a mass range from 500 to 5000 Da. The remaining parameters were kept as default settings. During the validation, MSBooster (version 1.3.17) was used for rescoring, and Percolator^39^ (version 3.7.1, default parameters) was used for PSM validation. FDR level was set to 1% for PSM, peptide, and protein. The identified proteins from the search formed a sample-specific protein database containing 97249 protein sequences. In addition, DDA raw files were subjected to de novo sequencing as previously described^1^. As a result, a total of 232863 protein sequences were included in the microbial database. This database was combined with the standard *Mus musculus* proteome (https://www.uniprot.org/proteomes/UP000000589, accessed on 2023-04-27) to process acquired datasets.

A schematic workflow for microbial database construction is summarized in Supplementary Fig. 7.

### Spectral library generation for DIA-NN analysis

The combined FASTA search space for benchmark comparison described above was used as input for library prediction in DIA-NN (v2.0.2) in a library-free mode based on in silico-predicted spectra. Peptides were generated by in silico digestion using Trypsin/P with up to two missed cleavages and N-terminal methionine excision enabled. Carbamidomethylation of cysteine was set as a fixed modification, while methionine oxidation, N-terminal acetylation, and heavy isotopic labeling of arginine (+10.0083 Da) and lysine (+8.0142 Da) were specified as variable modifications, allowing a maximum of two variable modifications per peptide. Peptide lengths were restricted to 7-30 amino acids. Precursor m/z values were limited to 250-1200 with charge states of 2-4, and fragment ions were considered in the range of 100-1700 m/z. DIA-NN’s deep learning models were used to predict fragment intensities, retention times, and ion mobility values. Proteotypicity was set to “Protein names (from FASTA),” and heuristic protein inference was disabled. Mass accuracy for MS1 and MS2 was set to automatic determination. The resulting predicted library comprised 12,412,855 precursors corresponding to 109,255 protein groups. To prevent incorrect retention-time alignment, DIA-NN searches were performed separately for each acquisition method and gradient length.

For the colonic samples presented in Fig. 4, pooled peptide samples (generated by combining all proximal or distal colon samples) were repeatedly analyzed using both DIA- and Slice-PASEF to monitor LC-MS performance and to support the generation of a sample-specific experimental spectral library. In addition, fractionated pooled samples were acquired in DIA-PASEF mode to further increase spectral coverage. These datasets were processed using DIA-NN (v2.3.0) with a predicted library comprising 26,374,776 precursors derived from a reduced microbial database (232,863 protein sequences), the standard *Mus musculus* proteome (UniProt UP000000589, accessed 2023-04-27), and DIA-NN contaminant sequences. Trypsin/P digestion was specified with a maximum of one missed cleavage, and N-terminal methionine excision was enabled. Carbamidomethylation of cysteine was used as a fixed modification, whereas methionine oxidation and N-terminal acetylation were allowed as variable modifications, permitting a maximum of one variable modification per peptide. Peptide length was restricted to 7–30 amino acids, precursor m/z values were set to 250–1200 with charge states of 2–3, and fragment ions were considered between 100 and 1700 m/z. Proteotypicity was set to “Isoform IDs,” and protein inference was disabled. The resulting experimental spectral library contained 184,884 precursors corresponding to 85,044 protein groups and was used for DIA- and Slice-PASEF analysis of individual samples.

For all searches conducted in DIA-NN, RT-dependent cross-run normalization and QuantUMS^40^ (high precision) options were selected for quantification. All DIA-NN search outputs were further processed with the R package, DIA-NN (https://github.com/vdemichev/diann-rpackage), to calculate the MaxLFQ^41^ quantitative intensities for all identified peptides and protein groups with *q*-value ≤ 0.01 as criteria at precursor and protein group levels.

A schematic workflow for data processing strategies used in the study is summarized in Supplementary Fig. 7.

### Sample preparation for PentaPASEF benchmarking

The human fecal background was generated by pooling fecal peptide samples produced in-house from multiple independent studies to ensure consistency and maximize proteomic complexity. The pooled fecal peptide mixture was diluted with 0.15% (w/v) n-dodecyl-β-D-maltoside (DDM; Sigma-Aldrich) to the desired concentrations.

*Ligilactobacillus murinus* (DSM 20452) and *Salinibacter ruber* (DSM 13855) were obtained from DSMZ (Braunschweig, Germany), and bacterial culture conditions were as previously reported^1^. Peptides derived from the two species were mixed at a fixed ratio of 1:3 (*L. murinus*:*S. ruber*) and subsequently diluted with 0.15% DDM to generate the required spike-in levels. Owing to the extended acquisition period, samples corresponding to different spike-in concentrations were further aliquoted and stored at −20 °C. For each acquisition method and LC gradient, a fresh aliquot was thawed immediately before analysis to minimize sample loss and variability associated with prolonged storage in the autosampler.

### Taxonomic and functional annotation and quantification

Taxonomic annotation was performed using the Unipept web application^42^ with default settings. Peptides present in the peptide quantification output of each method and gradient were submitted for annotation, and the resulting taxa assignments were merged back into the quantification matrix, where peptide intensities from acquisition triplicates were averaged. Taxa were considered confident when supported by at least three taxa-specific peptide entries, and taxa abundances were quantified by summing the intensities of taxa-assigned peptides. The microbial protein databases used in this manuscript were annotated using EggNOG-mapper^43^ (http://eggnog-mapper.embl.de/) with default settings to retrieve potential functions and pathways. The annotated functions were merged with the peptide quantification matrix using Meta4P^44^. The quantification of a functional entry was done by summing peptide intensities where the corresponding proteins were annotated to this functional entry.

For the in vivo dataset, peptides identified by DIA- and Slice-PASEF were annotated separately using Unipept and merged with the corresponding peptide quantification matrices. Confident taxa required at least three taxa-specific peptide entries, and taxa intensities were computed by summing peptide intensities after applying a content-wise detection filter, retaining peptides only if they were detected in all replicates of at least one group (colonic region × genotype × timepoint). The taxon-specific functions were analyzed using Meta4P with peptide quantification data from DIA-NN, confident taxonomic annotation, and functional annotation files from EggNOG-mapper as inputs. Quantification of taxon-specific functions was performed by summing the peptide intensities assigned to taxa and associated with specific functions.

### Calculation of relative species abundance rank

Species-level relative abundance ranks were calculated from taxonomically annotated peptide intensities. Species supported by at least three taxon-specific peptides were retained. For each sample, peptide intensities assigned to the same species were summed, and the species-level summed intensity was divided by the total summed intensity of all retained species to obtain relative abundance. Species were then ranked within each sample according to relative abundance, with rank 1 assigned to the most abundant species. For spike-in rank visualization, relative abundances were averaged across acquisition replicates for each method, gradient, and dilution level before ranking. For rank-variability analysis, ranks were calculated for each replicate sample, and the coefficient of variation of species ranks across spike-in replicates was used as a measure of compositional stability.

### Statistical analysis

Pairwise comparisons presented in Supplementary Fig. 4 were performed using a two-sided Wilcoxon rank-sum test (Mann–Whitney U test) in R (version 4.5.2). Differential abundance analysis of species, KEGG functions, species–KEGG associations, and host proteins (Fig. 4) was performed using the limma (version 3.66.0) framework. For each colonic content and time point, log₂-transformed abundances were modeled using linear models, genotype contrasts were tested using empirical Bayes–moderated t-statistics with robust variance estimation, and multiple testing correction was applied independently for each contrast within each colonic content and time point using the Benjamini-Hochberg procedure.

### Performance scoring for PentaPASEF

To summarize the benchmark results into a practical method-selection guide, performance scores were calculated for each acquisition method and LC gradient across representative analytical criteria, including identification depth, low-abundance sensitivity, quantitative precision, quantitative accuracy, species-level abundance consistency, and functional annotation depth/consistency. Two penalty strategies were applied before normalization. For qualitative recovery metrics, such as identification depth or feature detection, scores were adjusted by replicate variability to favor methods that combined high coverage with reproducible performance. For quantitative metrics, such as precision, abundance consistency, or functional reproducibility, scores were additionally adjusted for the number of quantified features to avoid assigning high scores to methods with low variability but limited coverage. Thus, methods were rewarded when they provided both stable quantification and sufficient analytical depth. Adjusted values were then normalized within each gradient to a 1-5 scale, with higher scores indicating better relative performance for the corresponding criterion.

### Reporting summary

Further information on research design is available in the Nature Portfolio Reporting Summary linked to this article.

## Supplementary information

Supplementary Information

Supplementary Data 1

Supplementary Data 2

Supplementary Data 3

Supplementary Data 4

Supplementary Data 5

Supplementary Data 6

Supplementary Data 7

Supplementary Data 8

Supplementary Data 9

Supplementary Data 10

Supplementary Data 11

Supplementary Data 12

Supplementary Data 13

Supplementary Data 14

Reporting Summary

Transparent Peer Review file

## Source data

Source Data

## Data Availability

The mass spectrometry proteomics data have been deposited to the ProteomeXchange Consortium via the PRIDE partner repository with the dataset identifiers PXD073779 and PXD073688. Source data are provided with this paper.

## Code availability

The code used for data processing, data filtering, and performance scoring in this study is publicly available and has been deposited in GitHub repository (https://github.com/CoEMetaproteomics) under the MIT License. The specific version of the code associated with this publication is archived in Zenodo (10.5281/zenodo.21157364)^45^.
