# Supplementary material for: Systematic evaluation of PASEF acquisition strategies in complex metaproteomes
Source: Nat Commun. 2026 Aug 3;17:7406. doi: 10.1038/s41467-026-75845-5 (PMC13434454; doi:10.1038/s41467-026-75845-5)
Supplement: Supplementary file 1 [file 41467_2026_75845_MOESM16_ESM.pdf]

nature portfolio

Corresponding author(s): David Gomez-Varela
Last updated by author(s): Jul 3, 2026

Reporting Summary

Nature Portfolio wishes to improve the reproducibility of the work that we publish. This form provides structure for consistency and transparency in reporting. For further information on Nature Portfolio policies, see our Editorial Policies and the Editorial Policy Checklist.

Statistics

For all statistical analyses, confirm that the following items are present in the figure legend, table legend, main text, or Methods section.

n/a	Confirmed	
☐	☒	The exact sample size (*n*) for each experimental group/condition, given as a discrete number and unit of measurement
☐	☒	A statement on whether measurements were taken from distinct samples or whether the same sample was measured repeatedly
☐	☒	The statistical test(s) used AND whether they are one- or two-sided<br>*Only common tests should be described solely by name; describe more complex techniques in the Methods section.*
☒	☐	A description of all covariates tested
☐	☒	A description of any assumptions or corrections, such as tests of normality and adjustment for multiple comparisons
☐	☒	A full description of the statistical parameters including central tendency (e.g. means) or other basic estimates (e.g. regression coefficient) AND variation (e.g. standard deviation) or associated estimates of uncertainty (e.g. confidence intervals)
☐	☒	For null hypothesis testing, the test statistic (e.g. *F*, *t*, *r*) with confidence intervals, effect sizes, degrees of freedom and *P* value noted<br>*Give P values as exact values whenever suitable.*
☒	☐	For Bayesian analysis, information on the choice of priors and Markov chain Monte Carlo settings
☒	☐	For hierarchical and complex designs, identification of the appropriate level for tests and full reporting of outcomes
☐	☒	Estimates of effect sizes (e.g. Cohen's *d*, Pearson's *r*), indicating how they were calculated

Our web collection on statistics for biologists contains articles on many of the points above.

Software and code

Policy information about availability of computer code

Data collection	The LC-MS data presented in the manuscript was newly generated.<br>The proteome fasta files were downloaded from public resources (detailed in Methods).
Data analysis	Software used in the manuscript along with the version information and parameters were described in the Methods section. Data filters/criteria were also listed in the Methods.<br>MSfragger (Version 4.1/4.3), FragPipe (Version 22.0/23.1), MSBooster (Version 1.2.31/1.3.17), Percolator (version 3.6.5/3.7.1), DIA-NN (version 2.0.2), R (version 4.5.2), BPS-Novor, DeepLC (version 2.2.27), IM2Deep (Version 0.1.7), Diamond (version 2.1.9).

For manuscripts utilizing custom algorithms or software that are central to the research but not yet described in published literature, software must be made available to editors and reviewers. We strongly encourage code deposition in a community repository (e.g. GitHub). See the Nature Portfolio guidelines for submitting code & software for further information.

Data

Policy information about availability of data

All manuscripts must include a data availability statement. This statement should provide the following information, where applicable:
- Accession codes, unique identifiers, or web links for publicly available datasets
- A description of any restrictions on data availability
- For clinical datasets or third party data, please ensure that the statement adheres to our policy

The mass spectrometry proteomics data have been deposited to the ProteomeXchange Consortium via the PRIDE partner repository with the dataset identifiers

PXD073779 and PXD073688. Source data are provided with this paper.

Research involving human participants, their data, or biological material

Policy information about studies with human participants or human data. See also policy information about sex, gender (identity/presentation), and sexual orientation and race, ethnicity and racism.

Reporting on sex and gender	Not applicable; We used human fecal peptides generated from other projects and pooled for this study to have a unified background.
Reporting on race, ethnicity, or other socially relevant groupings	Not applicable; We used human fecal peptides generated from other projects and pooled for this study to have a unified background.
Population characteristics	Not applicable; We used human fecal peptides generated from other projects and pooled for this study to have a unified background.
Recruitment	Not applicable; We used human fecal peptides generated from other projects and pooled for this study to have a unified background.
Ethics oversight	Original human fecal sampling from other projects carried out at the University of Vienna was under the ethical approval (01149).

Note that full information on the approval of the study protocol must also be provided in the manuscript.

Field-specific reporting

Please select the one below that is the best fit for your research. If you are not sure, read the appropriate sections before making your selection.

[x] Life sciences [ ] Behavioural & social sciences [ ] Ecological, evolutionary & environmental sciences

For a reference copy of the document with all sections, see nature.com/documents/nr-reporting-summary-flat.pdf

Life sciences study design

All studies must disclose on these points even when the disclosure is negative.

Sample size	Sample size is not relevant to the results presented in Figure 1-3, where only technical replicates were used.<br>For the colon injury model used for Figure 4, six mice were included in each condition. No statistical analysis was carried out to determine sample size. Based on our previous experiments and published studies, we included 6 animals per group for Fig.4, in order to comply with "3R" rules limiting the number of animals used in research, while reaching an adequate number of animals to avoid under-powering the study and being able to draw statistically significant conclusions.
Data exclusions	No data was excluded.
Replication	Sample analysis related to Figure 1-3 were done in spike-in triplicates and acquisition triplicates at LC-MS level.
Randomization	Mice used in Fig.4 were allocated into experimental groups based on different genotypes. The data acquisition related to Figure 4 was randomized for LC-MS analysis.
Blinding	Blinding was relevant to the study, as the investigators prepared samples according to the experimental design.

Reporting for specific materials, systems and methods

We require information from authors about some types of materials, experimental systems and methods used in many studies. Here, indicate whether each material, system or method listed is relevant to your study. If you are not sure if a list item applies to your research, read the appropriate section before selecting a response.

Materials & experimental systems

n/a	Involved in the study	
[x]	[ ]	Antibodies
[x]	[ ]	Eukaryotic cell lines
[x]	[ ]	Palaeontology and archaeology
[ ]	[x]	Animals and other organisms
[x]	[ ]	Clinical data
[x]	[ ]	Dual use research of concern
[x]	[ ]	Plants

Methods

n/a	Involved in the study	
[x]	[ ]	ChIP-seq
[x]	[ ]	Flow cytometry
[x]	[ ]	MRI-based neuroimaging

Animals and other research organisms

Policy information about studies involving animals; ARRIVE guidelines recommended for reporting animal research, and Sex and Gender in Research

Laboratory animals	Mice for in vivo experiments (Fig. 4) were male and housed under specific pathogen-free (SPF) conditions according to the criteria of the Federation for Laboratory Animal Science Associations (FELASA) (12-hour light/dark cycles at 24–26°C) in the mouse facility at the Technical University of Munich (School of Life Sciences Weihenstephan). All mice received a standard diet (autoclaved V1124-300, Ssniff) ad libitum, autoclaved water and were sacrificed by CO2 or isoflurane.<br>Details of the animal models can be found in our previous study23. Briefly, Hsp60fl/fl mice and Hsp60fl/fl x VillinCreERT2-Tg mice were generated as described previously34 to create IEC-specific Hsp60 knockout mice via tamoxifen induction (Hsp60Δ/ΔIEC;Il10+/+). In addition, Hsp60fl/fl and Hsp60Δ/ΔIEC;Il10+/+ mice were crossed with whole body Il-10 knockout mice (Il10-/-) to generate Hsp60fl/fl;Il10-/- and Hsp60Δ/ΔIEC;Il10-/- mice23. For conditional Hsp60 deletion, mice and appropriate control mice (6-weeks of age) were kept on phytoestrogen-reduced diet 1005 (V1154-300, Ssniff) for four weeks under SPF conditions. Afterwards, mice received 400mg tamoxifen citrate per kg chow feed (CreActive T400 (10mm, Rad), Genobios) ad libitum for 7 days. After the induction phase, tamoxifen diet was replaced with the phytoestrogen-reduced diet. During and after the induction phase, mice were monitored daily and aborted when a combined score considering weight loss, changes in stool consistency, general behaviour, and general state of health was reached. Animals were sacrificed at the indicated time points (D4, D8, and D14 after tamoxifen diet). All mice and their respective genotypes were generated and maintained on an in-house crossing of C57Bl/6N and C57Bl/6J background.<br><br>Ligillacotobacillus murinus (DSM 20452, L. murinus) and Salinibacter ruber (DSM 13855, S. ruber) were purchased from DSMZ (Braunschweig, Germany). Bactrial culture is described in our previous study (https://doi.org/10.1038/s41467-025-61977-7).
Wild animals	Not applicable
Reporting on sex	Mice used in the in vivo experiments (Fig. 4) were only male animals.
Field-collected samples	Not applicable
Ethics oversight	Human fecal sampling carried out at the University of Vienna was under the ethical approval (01149). Mouse work carried out at the Technical University of Munich, as well as maintenance and breeding of mouse lines, were approved by the Committee on Animal Health Care and Use of the state of Upper Bavaria (Regierung von Oberbayern; AZ ROB-55.2-2532.Vet_02-14-217, AZ ROB-55.2-2532.Vet_02-20-58, AZ ROB-55.2-2532.Vet_02-18-37) and performed in strict compliance with the EEC recommendations for the care and use of laboratory animals (European Communities Council Directive of November 24, 1986 (86/609/EEC)).

Note that full information on the approval of the study protocol must also be provided in the manuscript.

Plants

Seed stocks	Not applicable
Novel plant genotypes	Not applicable
Authentication	Not applicable
