# Supplementary material for: Systematic evaluation of PASEF acquisition strategies in complex metaproteomes
Source: Nat Commun. 2026 Aug 3;17:7406. doi: 10.1038/s41467-026-75845-5 (PMC13434454; doi:10.1038/s41467-026-75845-5)
Supplement: Supplementary file 2 [file 41467_2026_75845_MOESM17_ESM.pdf]

nature portfolio

Peer Review File

Systematic evaluation of PASEF acquisition strategies in complex metaproteomes

Corresponding Author: Dr David Gomez-Varela

This file contains all reviewer reports in order by version, followed by all author rebuttals in order by version.

Version 0:

Reviewer comments:

Reviewer #1

(Remarks to the Author)
The manuscript presents a comparison of different PASEF acquisition strategies applied to very complex metaproteomic samples. The study is clearly well planned and stands out for both its scale and the consistency of the experimental design. The combination of spike-in experiments across different gradients and input levels, together with the in vivo validation, adds real value to the dataset. Overall, it is evident that a substantial amount of work has gone into this study, and it addresses a question that is becoming increasingly relevant in the field. I would suggest a minor revision.
General comments:
While the work is solid, it still feels mainly like a benchmarking study and does not fully explore its broader implications. The comparisons are detailed and technically sound, but the overall message is not always immediately clear. In particular, it would be useful to guide the reader more explicitly on when one acquisition strategy should be preferred over another. As it stands, the conclusions are correct, but a bit cautious, as the differences between methods are described without being fully translated into practical recommendations.
Detailed comments:
Regarding the data analysis workflow, the use of different pipelines for DDA and DIA approaches is understandable, but it makes the interpretation less straightforward. It is not always easy to distinguish whether the observed differences come from the acquisition strategies themselves or from the software used. This point would deserve a more explicit discussion. If possible, even a limited cross-validation would help to strengthen the conclusions.
Concerning the spike-in design, it is well controlled and appropriate for benchmarking purposes. However, the use of only two bacterial species inevitably simplifies the system. This does not invalidate the results, but it does suggest that conclusions about sensitivity and quantitative performance should be expressed with some caution, especially when extending them to more complex, real-world microbiomes. It might be useful if the authors could comment on this more clearly.
The in vivo part is, in my opinion, one of the strongest aspects of the study. The agreement between DIA- and Slice-PASEF is convincing and supports the main conclusions. That said, the biological interpretation could be developed a bit further. The pathways and host responses are reported, but they are not fully connected into a clear mechanistic picture. Even a short effort in this direction would make this section more impactful.
A final point concerns Slice-PASEF. It clearly emerges as one of the best-performing approaches, and this is well supported by the data. However, its practical limitations are only briefly mentioned. Aspects such as larger data size and higher computational demands are important in practice, and a more balanced discussion would be helpful for readers.
Overall, this is a well-executed and technically solid study that will be of interest to the metaproteomics community. With some adjustments, especially in the Discussion, the work could make a valuable contribution to the field.

Reviewer #2

(Remarks to the Author)
Xian et al present a systematic and large scale benchmarking of five PASEF acquisition strategies using a complex fecal metaproteome background with controlled spike ins. This study fills a gap in the field by providing a matched, side by side comparison of modern PASEF modes under high complexity metaproteomic conditions. The scale of the work is exceptional - 540 LC–MS runs performed under carefully harmonized conditions, enabling a rigorous evaluation.

The spike in design is compelling, offering a realistic test that highlights the strengths of DIA and Slice-PASEF in sensitivity,

reproducibility, and functional depth. The authors further validate these two top performing methods in a murine colonic injury model, demonstrating that acquisition strategy meaningfully influences downstream biological interpretation. This connection between technical optimization and biological insight is noteworthy.

The writing is clear, the analyses are thorough, and the conclusions are well supported by the data. The integration of functional and taxonomic perspectives adds substantial value for the metaproteomics community. The authors' discussion of current limitations positions it as a foundational resource for future methodological development.

Overall, this is a strong, timely, and highly relevant contribution. However, addressing the following points with respect to methodological clarifications and interpretive nuances will further strengthen the manuscript and broaden its impact.

1. The benchmark design is strong, but the rationale for certain acquisition parameters requires further expansion to clarify potential issues. The authors acknowledge that acquisition windows, duty cycles, and isolation schemes were not systematically tuned, which raises critical questions regarding how sensitive the final conclusions are to these settings. Specifically, it is unclear whether the lower performance of midia-PASEF stems from suboptimal windowing rather than inherent method limitations. Additionally, the decision to evaluate Slice-PASEF using only a one-frame configuration after initial testing needs a more justification. To address these concerns, the authors should provide a supplementary table summarizing all acquisition parameters and include a brief discussion on how deviations from optimal settings might influence the performance ceiling of each evaluated method.
2. For Figure 3B observations, can the author's comment on the use of DIA-NN's quantitation under extreme dynamic range conditions? Can alternative quantification methods be explored and would they yield similar conclusions?
3. While, the biological validation is compelling, can the authors more clearly articulate why the murine colonic injury model is representative of typical metaproteomic challenges?
4. While the comparison between DDA and DIA pipelines is technically sound, the authors rely on MSFragger/FragPipe for DDA and DIA-NN for DIA-based methods. Given that these platforms employ distinct algorithms, it is unclear how much of the performance gap particularly the higher unique identifications in DDA-PASEF is driven by the search engine rather than the acquisition mode. To clarify these pipeline-dependent effects, the authors should state whether they cross-validated the data, such as processing the DIA-PASEF data through the FragPipe DIA workflow.
5. Since the authors have used Spectronaut 20 for their confirmatory analysis (Lines 145-146) , I wonder why that was not used as an algorithm of choice?
6. Consider defining each PASEF mode in a short table for readers unfamiliar with Slice/Synchro/miDIA.
7. Fig. 2F and Fig. 2G are excellent but dense. Consider adding small schematic icons to help readers interpret these figures.
8. Fig. 4C–D: The upward shift in Slice-PASEF intensities is important; consider quantifying the median shift.
9. The microbial database construction section is detailed but could benefit from a schematic summarizing the multi-step database generation.
10. To maximize the utility of this work for the metaproteomics community, the authors should consider including a summary table which enlists the benefits of the five 'pentaPASEF' acquisition flavors for metaproteomics. This table can clearly delineate the specific benefits, trade-offs, and ideal use cases for each method based on the results of this study. This would provide a valuable decision-making roadmap for researchers selecting the most appropriate strategy for complex microbiome samples.
11. On Line 198, authors mention "few lower correlations observed for Slice-PASEF at 22 min ($r < 0.9$)" did you mean 45 min and not 22 mins?
12. A few typos appear ("sassword" on line 665 and Acknoledgements on line 770). Please correct.

Reviewer #3

(Remarks to the Author)
The manuscript entitled "Systematic Evaluation of PASEF Acquisition Strategies in Complex Metaproteomes" presents a benchmarking study of different mass spectrometry data acquisition strategies for characterizing gut microbiome metaproteomes from identification, quantification, species-level, and functional perspectives.

General Comments:
The manuscript is well written, and the methods are generally sound. While some of the results were expected, if not already known (e.g., DDA vs. DIA), others are interesting, particularly the differences observed from the DIA-based methods. From an analytical standpoint, the benchmarking is fairly comprehensive. Nevertheless, I do have some comments regarding how some results are presented and discussed, as well as the lack of comprehensiveness from a computational perspective.

Major Comments:
1. The fact that DDA and DIA workflows were not analyzed using the same metaproteome sequence database (database vs. library) is problematic. Why not use the library generated for the DIA-NN analysis to also perform the DDA analysis? This would reduce an additional source of variability that could contribute to the observed differences. This is somewhat akin to comparing apples and oranges.

2. Another missing aspect of this paper is the impact of the spectral library itself. Metaproteome spectral library generation is an important area of research, and it has been shown that the choices made during library generation can substantially affect the final results. A comparison of different library sizes (large versus more focused libraries) would therefore be important.

3. Why require 3 taxa-specific peptides for taxon identification? A single taxa-specific peptide should be sufficient for

inclusion in the analysis.

4. In Figure 3, it is unclear how the rank of a microbe is calculated. This should be clarified in the Methods.

5. In Figure 4B, why was the PCA analysis not performed directly on peptide or protein identifications or quantifications? In fact, this analysis should be performed for benchmarking shown in Figure 2. This would allow for a better visualization of the differences among acquisition strategies. Furthermore, the Figure 4B caption is not descriptive enough.

Minor Comments:
6. Starting at line 156: "Nevertheless, DIA-based strategies retained higher recovery across gradients, demonstrating superior detection of low-abundance peptides in complex matrices." This statement is not true under all conditions.

7. Figure 2F is acceptable, but showing the distribution of the number of peptides supporting each species, whether shared or unique, would be a much more effective way to present the data. To simplify the presentation, replicates could be pooled into one distribution.

8. At line 202, the authors state: "DDA-PASEF displayed a pronounced gradient dependence, with variability decreasing at longer separations, whereas DIA-based methods were less affected by gradient length." The average CVs should be provided in the text.

9. At line 320, the term "borderline statistical behavior" is overly optimistic. Results that fail multiple hypothesis correction are simply not statistically significant.

Version 1:

Reviewer comments:

Reviewer #1

(Remarks to the Author)
The authors have satisfactorily and comprehensively addressed the comments previously raised.

Reviewer #2

(Remarks to the Author)
The authors have answered my questions and have made the necessary changes. The manuscript is ready for publication in my opinion.

Reviewer #3

(Remarks to the Author)
The authors have addressed all of my comments.

Open Access This Peer Review File is licensed under a Creative Commons Attribution 4.0 International License, which permits use, sharing, adaptation, distribution and reproduction in any medium or format, as long as you give appropriate credit to the original author(s) and the source, provide a link to the Creative Commons license, and indicate if changes were made.
In cases where reviewers are anonymous, credit should be given to 'Anonymous Referee' and the source.
The images or other third party material in this Peer Review File are included in the article's Creative Commons license, unless indicated otherwise in a credit line to the material. If material is not included in the article's Creative Commons license and your intended use is not permitted by statutory regulation or exceeds the permitted use, you will need to obtain permission directly from the copyright holder.
To view a copy of this license, visit https://creativecommons.org/licenses/by/4.0/

Reviewer's Comments:

Reviewer #1 (Remarks to the Author)

The manuscript presents a comparison of different PASEF acquisition strategies applied to very complex metaproteomic samples. The study is clearly well planned and stands out for both its scale and the consistency of the experimental design. The combination of spike-in experiments across different gradients and input levels, together with the in vivo validation, adds real value to the dataset. Overall, it is evident that a substantial amount of work has gone into this study, and it addresses a question that is becoming increasingly relevant in the field. I would suggest a minor revision.

We thank the reviewer for the highly positive evaluation of our manuscript and for recognizing the scale, experimental consistency, and relevance of this study. We also appreciate the constructive suggestions provided below, which have helped us strengthen the manuscript. Regarding the comments raised by the reviewer, we provide point-by-point replies below.

General comments:

Comment 1: While the work is solid, it still feels mainly like a benchmarking study and does not fully explore its broader implications. The comparisons are detailed and technically sound, but the overall message is not always immediately clear. In particular, it would be useful to guide the reader more explicitly on when one acquisition strategy should be preferred over another. As it stands, the conclusions are correct, but a bit cautious, as the differences between methods are described without being fully translated into practical recommendations.

We thank the reviewer for this important and constructive comment. We agree that the manuscript should not only present a technical comparison, but should also translate the observed performance differences into practical guidance for the metaproteomics community.

To address this point, we added a new data-derived performance summary figure in the revised manuscript (Fig. R1; now included as Supplementary Fig. 3). For each acquisition strategy, we calculated normalized performance scores on a 1–5 scale across evaluation criteria covering identification depth, low-abundance sensitivity, quantitative precision and accuracy, and metaproteomics-specific readouts, including taxonomic and functional profiling. These scores were calculated directly from the benchmark data using mean performance values penalized by replicate variability, thereby incorporating both the magnitude and reproducibility of each metric. The scoring procedure is now described in the Methods section (lines 815–829 in the revised manuscript).

This analysis provides a practical overview of the relative strengths and trade-offs of each acquisition strategy. Consistent with the main results, DIA-based approaches achieved higher

overall scores than DDA-PASEF across most analytical dimensions, with DIA- and Slice-PASEF showing the strongest overall performance. We have also added a new Results/Discussion section to explicitly translate these findings into practical recommendations (lines 273-283, lines 459-478 in the revised manuscript). Specifically, we now clarify that acquisition strategy selection should depend on the experimental objective: DIA-PASEF provides a balanced option for routine and scalable metaproteomic profiling; Slice-PASEF is advantageous when maximum sensitivity and functional depth are prioritized and if longer gradients, storage space, and computational resources are not a limitation; DDA-PASEF remains useful for the generation of reduced microbial databases; and Synchro- and midia-PASEF may benefit from further acquisition-parameter optimization in complex metaproteomic settings.

We believe these revisions make the broader message of the study clearer and provide readers with a more actionable framework for selecting PASEF acquisition strategies according to experimental goals, sensitivity requirements, quantitative robustness, throughput, and computational resources.

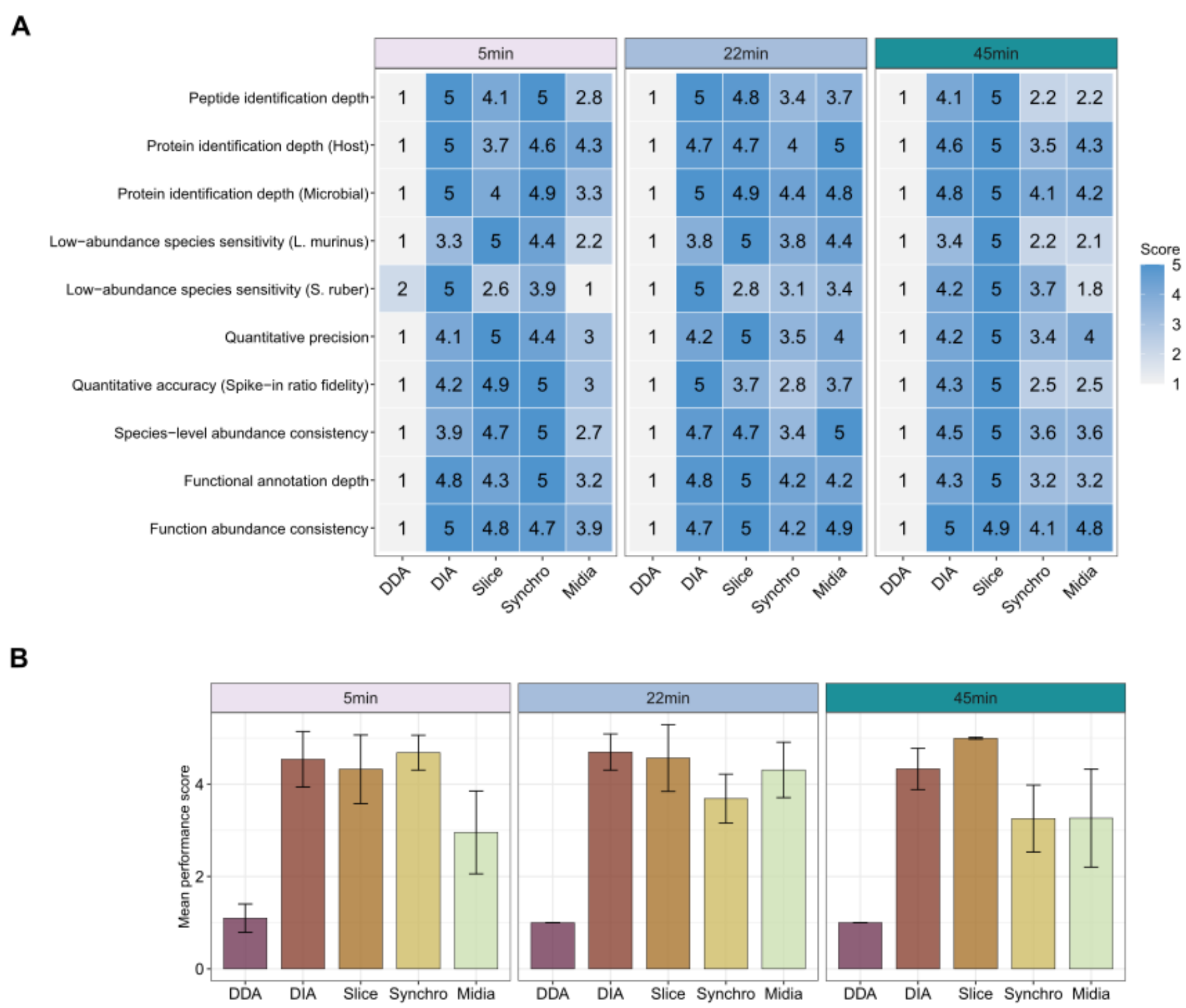

Fig. R1: Data-derived practical guidance for PentaPASEF method selection. A, Normalized performance scores for five PASEF acquisition strategies across gradients and analytical evaluation criteria. Scores range from 1 to 5, with higher values indicating better performance. B, Mean performance score across evaluation criteria for each acquisition strategy and gradient. Bars indicate the mean ± SD across the scored criteria.

Detailed comments:

Comment 2: Regarding the data analysis workflow, the use of different pipelines for DDA and DIA

approaches is understandable, but it makes the interpretation less straightforward. It is not always easy to distinguish whether the observed differences come from the acquisition strategies themselves or from the software used. This point would deserve a more explicit discussion. If possible, even a limited cross-validation would help to strengthen the conclusions.

We thank the reviewer for raising this important point. We agree that the use of different software pipelines for DDA- and DIA-based acquisitions could potentially confound the interpretation of acquisition-dependent performance differences. Our original workflow used FragPipe/MSFragger for DDA-PASEF and DIA-NN for DIA-based methods because these platforms are widely used, freely available for academic use, and well established for their respective acquisition types.

To directly address the reviewer's concern, we performed an additional reciprocal cross-validation analysis. Specifically, DIA-PASEF data were reprocessed using the FragPipe/diaTracer workflow, and DDA-PASEF data were reprocessed using DIA-NN v2.3.2, which recently introduced support for DDA data analysis. The resulting outputs were subjected to the same filtering strategy used in the manuscript. The peptide identification summary is shown in Fig. R2 and has been added to the revised manuscript as Supplementary Fig. 6a.

As expected, absolute identification numbers differed between software platforms, reflecting differences in search algorithms and scoring strategies. DIA-NN generally yielded higher peptide identifications than FragPipe, including DDA-PASEF. However, the major acquisition-dependent trend remained unchanged: DIA-PASEF outperformed DDA-PASEF in both software environments in terms of average peptide identifications and reproducibility, particularly under the 5-min gradient condition. In addition, the saturation-like behavior observed for DIA-based acquisitions at 22–45 min was also captured when DIA-PASEF data were processed with FragPipe, whereas DDA-PASEF continued to show a stronger gradient-dependent increase in identifications.

These results indicate that software choice influences absolute identification depth but does not explain the primary performance hierarchy observed between DDA- and DIA-based acquisition strategies. We have revised the Discussion (lines 480-492 in the revised manuscript) to clarify this point and to more explicitly acknowledge the contribution of both acquisition mode and software ecosystem to metaproteomic data analysis.

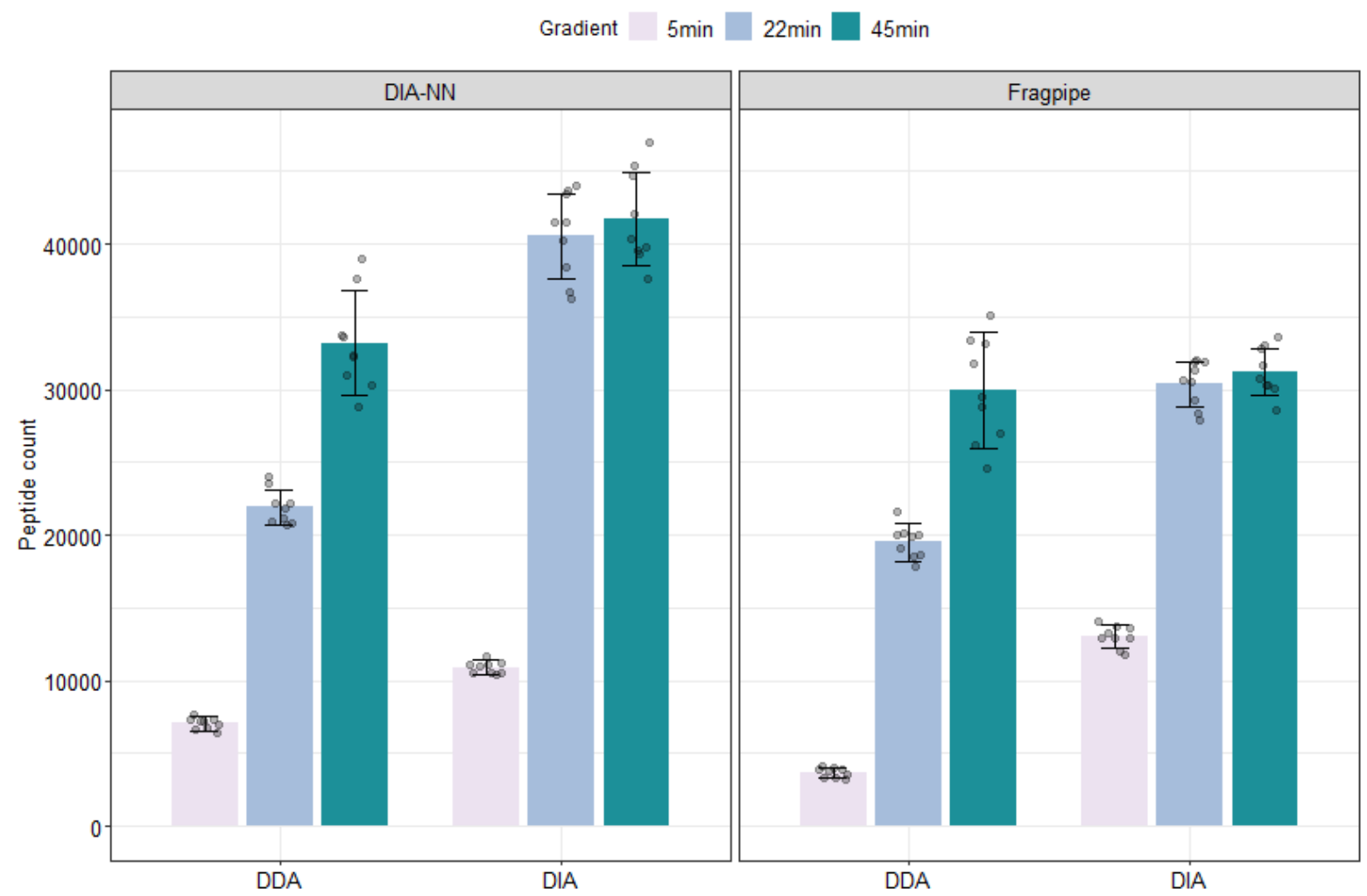

Fig. R2: Number of identified peptides for DDA- and DIA-PASEF analyzed with FragPipe and DIA-NN. The bars show the average number of peptides (n=9 replicates), and the error bars represent the standard deviations.

Comment 3: Concerning the spike-in design, it is well controlled and appropriate for benchmarking purposes. However, the use of only two bacterial species inevitably simplifies the system. This does not invalidate the results, but it does suggest that conclusions about sensitivity and quantitative performance should be expressed with some caution, especially when extending them to more complex, real-world microbiomes. It might be useful if the authors could comment on this more clearly.

We thank the reviewer for raising this important point. We agree that using two bacterial reference species does not capture the full taxonomic and biochemical diversity of natural microbiomes. However, the purpose of the spike-in experiment was not to generate a microbiome-wide reference community, but to introduce defined, traceable low-abundance targets into a highly complex fecal peptide background in order to isolate acquisition-dependent performance. This design allowed us to evaluate sensitivity, reproducibility, and ratio preservation under controlled conditions while maintaining realistic peptide interference from the fecal metaproteome.

Generating a truly microbiome-wide spike-in control is challenging because microbial communities differ substantially across body sites, environments, and disease states, and because absolute detectability depends on organism-specific properties such as cell size, proteome size, lysis and extraction efficiency, digestion efficiency, peptide ionization, and database completeness. A larger multi-species spike-in mixture would therefore introduce additional biological and technical variables that could obscure the acquisition-specific effects we aimed to test. By contrast, the use of two well-defined reference bacteria enabled controlled dilution, known ratios, and clear interpretation of low-abundance detection across acquisition methods.

We have revised the Discussion (lines 506-519 in the revised manuscript) to clarify that the spike-in data should be interpreted as a controlled peptide-level benchmark for relative method comparison, rather than as an absolute microbiome-wide sensitivity estimate. We also note that this design complements our previous intact-cell spike-in study (doi.org/10.1038/s41467-025-61977-7), in which *L. murinus* and *S. ruber* were added directly to fecal material to evaluate organism-level detection and quantification limits in the presence of extraction and digestion effects. Together, these approaches distinguish acquisition-dependent performance from organism- and sample-processing-dependent sensitivity limits.

Comment 4: The in vivo part is, in my opinion, one of the strongest aspects of the study. The agreement between DIA- and Slice-PASEF is convincing and supports the main conclusions. That said, the biological interpretation could be developed a bit further. The pathways and host responses are reported, but they are not fully connected into a clear mechanistic picture. Even a short effort in this direction would make this section more impactful.

We thank the reviewer for this constructive suggestion. We agree that the biological interpretation can be made clearer and better connected across microbial functional remodeling and host responses. In the original manuscript, we focused primarily on the concordance between DIA- and Slice-PASEF as a validation of acquisition-strategy robustness. In the revised manuscript, we have expanded this section to provide a clearer biological synthesis of the shared host–microbiome signals recovered by both methods.

To this end, we added a new microbial functional analysis summarizing significantly regulated microbial KEGG pathways in the Hsp60$^{\Delta/\Delta IEC}$;Il10$^{-/-}$ versus Hsp60$^{fl/fl}$;Il10$^{-/-}$ contrast to support the observations in host injury status (new Supplementary Fig. 5). Specifically, we only retained functional pathways that passed the significance threshold (adjusted p-value <= 0.05) in both DIA- and Slice-PASEF to ensure the confidence of biological interpretation. These pathways were further annotated to specific BRITE categories. This analysis shows sustained microbial functional remodeling over time and across colonic regions, including persistent changes in core metabolic categories and region-specific trajectories in signal transduction and glycan biosynthesis/metabolism. We now connect these microbial functional patterns to the host-proteome response. The distal colon retained stronger inflammatory and antimicrobial features at D14, including Reg-family proteins and S100a9, whereas the proximal colon showed stronger repair- and resolution-associated features, including Anxa1 and wound-healing-related proteins (lines 384-420 in the revised manuscript).

Comment 5: A final point concerns Slice-PASEF. It clearly emerges as one of the best-performing approaches, and this is well supported by the data. However, its practical limitations are only briefly mentioned. Aspects such as larger data size and higher computational demands are important in practice, and a more balanced discussion would be helpful for readers.

We thank the reviewer for this important point. We acknowledge that the superior analytical performance of Slice-PASEF should be discussed along with its practical costs. In the original

manuscript, we briefly noted (previous version lines 296-298) that Slice-PASEF generated substantially larger raw files and required longer DIA-NN processing times compared to DIA-PASEF data under the same data processing settings, including search space, RAM, and CPU allocation. In the revised manuscript, we have expanded this discussion and now describe more explicitly the trade-off between analytical depth and computational burden.

Specifically, we clarify that Slice-PASEF provides the highest sensitivity and quantitative depth in our benchmark, particularly for low-abundance microbial features and functional annotations, but this comes at the cost of increased raw data volume, longer processing time, and greater data-management requirements. We therefore now recommend Slice-PASEF primarily for studies where maximum sensitivity and functional depth are prioritized, such as low-input samples, rare taxa detection, or deep profiling of selected cohorts. In contrast, DIA-PASEF may represent a more practical default for larger studies where robust quantification, scalability, and computational efficiency are important considerations.

We have incorporated this point into the Discussion of the revised manuscript (lines 443-453): "*Although Slice-PASEF achieved the strongest overall analytical performance in this study, this gain should be interpreted together with its practical costs. Compared with DIA-PASEF under identical processing settings, including search space, RAM, and CPU allocation, Slice-PASEF generated substantially larger raw files and required longer DIA-NN processing times (Supplementary Fig. 4f). These increases are consistent with the denser MS/MS sampling intrinsic to the Slice acquisition scheme. Thus, while Slice-PASEF provides clear advantages in sensitivity and quantitative depth, its deployment requires greater storage capacity, processing time, and data-management resources. These practical considerations are particularly relevant when scaling metaproteomic analyses to large cohorts or high-throughput study designs.*"

Comment 6: Overall, this is a well-executed and technically solid study that will be of interest to the metaproteomics community. With some adjustments, especially in the Discussion, the work could make a valuable contribution to the field.

We thank the reviewer for this encouraging assessment and for recognizing the technical quality and relevance of the study, more importantly, for the constructive suggestions and comments.

Reviewer #2 (Remarks to the Author)

Xian et al present a systematic and large scale benchmarking of five PASEF acquisition strategies using a complex fecal metaproteome background with controlled spike ins. This study fills a gap in the field by providing a matched, side by side comparison of modern PASEF modes under high complexity metaproteomic conditions. The scale of the work is exceptional - 540 LC–MS runs performed under carefully harmonized conditions, enabling a rigorous evaluation.

The spike in design is compelling, offering a realistic test that highlights the strengths of DIA and Slice-PASEF in sensitivity, reproducibility, and functional depth. The authors further validate these two top performing methods in a murine colonic injury model, demonstrating that acquisition strategy meaningfully influences downstream biological interpretation. This connection between technical optimization and biological insight is noteworthy.

The writing is clear, the analyses are thorough, and the conclusions are well supported by the data. The integration of functional and taxonomic perspectives adds substantial value for the metaproteomics community. The authors' discussion of current limitations positions it as a foundational resource for future methodological development.

Overall, this is a strong, timely, and highly relevant contribution. However, addressing the following points with respect to methodological clarifications and interpretive nuances will further strengthen the manuscript and broaden its impact.

We sincerely thank the reviewer for this very positive and encouraging assessment of our manuscript. We also appreciate the constructive suggestions for strengthening the methodological clarity and the broader impact of the study. We provide point-by-point responses below.

Comment 1: The benchmark design is strong, but the rationale for certain acquisition parameters requires further expansion to clarify potential issues. The authors acknowledge that acquisition windows, duty cycles, and isolation schemes were not systematically tuned, which raises critical questions regarding how sensitive the final conclusions are to these settings. Specifically, it is unclear whether the lower performance of midia-PASEF stems from suboptimal windowing rather than inherent method limitations. Additionally, the decision to evaluate Slice-PASEF using only a one-frame configuration after initial testing needs a more justification. To address these concerns, the authors should provide a supplementary table summarizing all acquisition parameters and include a brief discussion on how deviations from optimal settings might influence the performance ceiling of each evaluated method.

We thank the reviewer for raising this important point. We agree that acquisition-parameter choices can affect the performance ceiling of each PASEF strategy, particularly for newer acquisition modes such as Slice- and midia-PASEF.

In the present study, our goal was to compare practically deployable implementations of the five PASEF modes under matched metaproteomic conditions, rather than to exhaustively optimize every possible parameter combination for each method. The acquisition methods used here were selected based on factory recommendations, published implementations, and preliminary tests on related samples. We had already provided a supplementary table summarizing the relevant acquisition parameters for all PASEF modes, including acquisition windows, duty cycle–related settings, and isolation schemes (Supplementary Data 13 in previous submission). In addition, all raw data have been deposited to the ProteomeXchange Consortium (PXD073779), where the specific acquisition methods can be accessed.

As the reviewer noted, we performed exploratory optimization for selected parameters expected to influence performance in our setup. Specifically, we evaluated Slice-PASEF frame densities and midia-PASEF window overlap configurations, and selected the one-frame Slice-PASEF configuration and 5-Da midia-PASEF overlap because these settings yielded the highest identifications in our preliminary comparison (Supplementary Fig. 1b). For Slice-PASEF, this observation is consistent with the original Slice-PASEF study, in which the one-frame scheme produced higher precursor and protein group identifications than the two-frame scheme in a low-input setting using 10 ng of K562 whole-proteome tryptic digest standard (doi.org/10.1101/2022.10.31.514544; Fig. 2 of the referenced study). Thus, the Slice-PASEF configuration used here was supported by both our preliminary tests and published evidence in a comparable low-input context.

For midia-PASEF, we agree that the relatively lower performance observed here should not be interpreted as an inherent limitation of the acquisition concept. The original midia-PASEF benchmark (doi.org/10.1101/2023.01.30.526204) was performed under substantially different conditions, including higher-input (e.g., 100 ng) HeLa digest samples and a different instrument platform (timsTOF Pro 2), and there is no published guidance for adapting midia-PASEF window designs to low-input fecal metaproteomes. Moreover, the original benchmarking primarily evaluated a specific midia windowing scheme (quadrupole isolation window widths of 36 Th in each frame and 24 Th overlap between two neighbouring frames). Alternative window widths, overlaps, duty cycles, or cycle designs may therefore further improve midia-PASEF performance in metaproteomic applications.

We have revised the Discussion (lines 547-564) to clarify that as: *"Our benchmark was designed to compare practically deployable implementations of each PASEF strategy under matched metaproteomic conditions, rather than to exhaustively optimize the full acquisition-parameter space of every method. Accordingly, the results should be interpreted as a comparison of the tested implementations and not as absolute performance ceilings for each acquisition concept. For Slice-PASEF, the one-frame configuration was selected based on our preliminary evaluation and is consistent with the original Slice-PASEF report, in which one-frame acquisition provided higher precursor and protein group identifications than two-frame acquisition under low-input conditions. By contrast, the relatively lower performance of midia-PASEF observed here should be interpreted as specific to the tested low-input fecal metaproteomic implementation. The original midia-PASEF benchmark was performed under substantially different conditions, including higher-input HeLa*

digest samples and a different instrument platform, and there is no published *guidance for adapting midia-PASEF window schemes to low-input metaproteomic samples. Alternative window widths, overlaps, duty cycles, cycle designs, and dedicated processing strategies may therefore improve midia-PASEF performance in complex metaproteomic applications."*

Comment 2: For Figure 3B observations, can the author's comment on the use of DIA-NN's quantitation under extreme dynamic range conditions? Can alternative quantification methods be explored and would they yield similar conclusions?

We thank the reviewer for raising this important point. We agree that quantitative accuracy under broad dynamic range and high-interference conditions can be affected by both acquisition strategy and software-specific quantification methods. DIA-NN was used for the main analysis because it provides a mature and widely adopted workflow for DIA-PASEF-type data, allowing consistent processing of DIA-, Slice-, Synchro- and midia-PASEF datasets within a unified framework.

For Fig. 3b, the expected high-to-medium spike-in ratio is 10-fold, which is within a commonly used range for proteomic quantification. We note that the high-to-low comparison would correspond to a 100-fold difference; however, because of the limited number of quantified peptides at the lowest spike-in level, this comparison was not suitable for robust assessment of quantitative accuracy. Even for the 10-fold comparison, the spike-in peptides were measured within a highly complex fecal peptide background and included low-abundance features approaching the detection limit, particularly under short-gradient conditions. Under such conditions, ratio compression can arise from multiple factors, including co-eluting background signals, low signal-to-noise ratios, interference during peak extraction, missingness, normalization effects, and peptide-specific ionization behavior. After reviewing the related literature, we did not find benchmark data evaluating quantification algorithms across such a broad dynamic range (e.g., 100-fold or higher). This was also confirmed in a discussion with Prof. Vadim Demichev, developer of DIA-NN. The main reasons pointed out by Prof. Demichev relate to the use of ground-truth dynamic range differences that reflect typical biological variation, as well as the assumption that biological experiments aiming to identify meaningful differences should use consistent peptide input amounts across samples whenever possible, to minimize artifacts introduced by extreme sample-to-sample variation.

To assess whether the ratio results presented in Fig. 3b were specific to DIA-NN quantification, we performed an additional confirmatory analysis using Spectronaut 20 for representative DIA-based datasets, including DIA- and midia-PASEF. The Spectronaut-based analysis reproduced the main qualitative trends observed with DIA-NN (Fig. R3 below): background peptide ratios were largely centered near zero, and *S. ruber* spike-in peptides showed ratio distributions shifted toward the expected 10-fold difference, with improved ratio preservation at longer gradients. Interpretation of *L. murinus* in the Spectronaut analysis was more limited because fewer *L. murinus* peptides were quantified, resulting in sparse distributions. Nevertheless, the results for background and *S.*

ruber support that the main conclusions from Fig. 3b are not specific to the DIA-NN quantification algorithm.

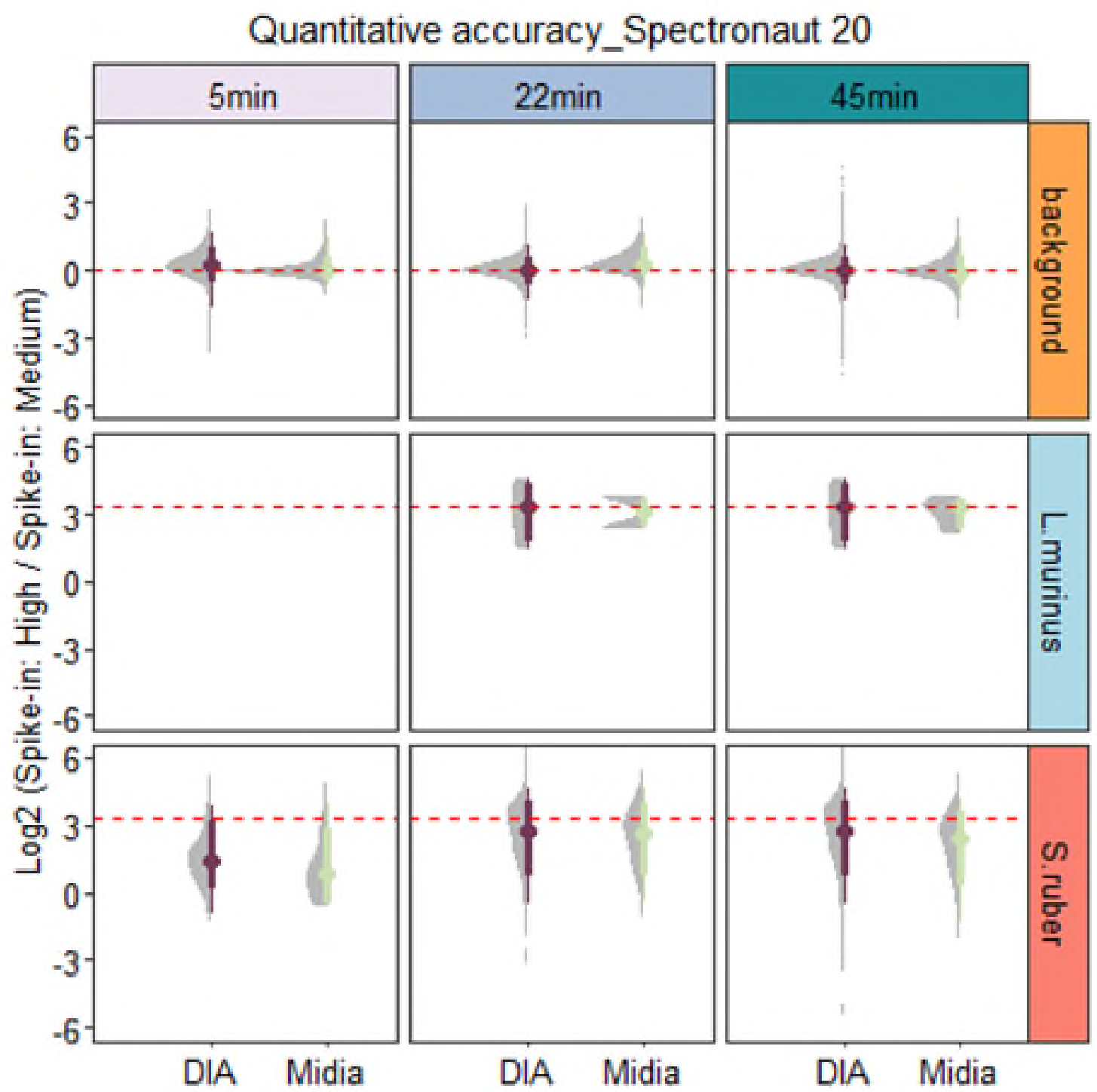

Fig. R3: Evaluation of quantitative accuracy using Spectronaut 20 quantification for DIA- and midia-PASEF datasets.

We have added this comparison as a new Supplementary Fig. 2c in the revised manuscript (lines 231-235): *"Additional Spectronaut 20 analysis of representative DIA- and midia-PASEF datasets reproduced the main quantitative accuracy trends observed with DIA-NN for background and S. ruber peptides, while interpretation of L. murinus was limited by the lower number of quantified spike-in peptides (Supplementary Fig. 2c)."*

Comment 3: While, the biological validation is compelling, can the authors more clearly articulate why the murine colonic injury model is representative of typical metaproteomic challenges?

We thank the reviewer for this helpful suggestion. We selected this model because colonic luminal samples contain a complex mixture of microbial proteins together with host-derived proteins released from the intestinal epithelium and immune compartment. Thus, they recapitulate a central analytical challenge in metaproteomics: quantifying microbial community functions in the presence of host proteins, broad dynamic range, and substantial biological variability. The MGnify mouse gut catalogue contains 112,951 genomes represented by 2,847 species, compared with 289,232 genomes and 4,744 species representatives in the human gut catalogue (https://www.ebi.ac.uk/metagenomics/browse/genomes). Although these catalogue sizes are derived from metagenomic data and do not directly define proteomic detectability, they illustrate

that the murine gut microbiome provides a taxonomically diverse and complex microbial background for evaluating metaproteomic acquisition strategies.

In addition, mouse models are among the most widely used systems for preclinical studies. The epithelial injury model introduces time-dependent and region-specific host–microbiome changes, including altered microbial functions and host responses related to epithelial repair, antimicrobial defense, inflammation, and hemostasis. Importantly, this model is accompanied by histological staining data published previously (doi.org/10.1016/j.chom.2024.06.013), which provide orthogonal tissue-level evidence of epithelial injury and recovery, offer biological context for interpreting the metaproteomic signatures, and help assess whether DIA- and Slice-PASEF recover consistent host–microbiome response patterns.

Comment 4: While the comparison between DDA and DIA pipelines is technically sound, the authors rely on MSFragger/FragPipe for DDA and DIA-NN for DIA-based methods. Given that these platforms employ distinct algorithms, it is unclear how much of the performance gap particularly the higher unique identifications in DDA-PASEF is driven by the search engine rather than the acquisition mode. To clarify these pipeline-dependent effects, the authors should state whether they cross-validated the data, such as processing the DIA-PASEF data through the FragPipe DIA workflow.

We thank the reviewer for raising this important point. This concern is closely related to the pipeline-comparability issue raised by Reviewer #1, Comment 2, and we have addressed it by performing reciprocal cross-platform validation analyses. Specifically, DIA-PASEF data were reprocessed using the FragPipe diaTracer workflow, and DDA-PASEF data were reprocessed using DIA-NN v2.3.2, which recently introduced support for DDA data analysis. The cross-platform analysis (Fig. R2) showed that peptide identification numbers differed between software platforms, with DIA-NN yielding modestly higher identifications (averaged among replicates) for DDA-PASEF than FragPipe. Importantly, the major acquisition-dependent trend was preserved: DIA-PASEF outperformed DDA-PASEF in both software environments, particularly at the 5-min gradient. This indicates that software choice influences absolute identification depth but does not account for the overall performance gap between DDA- and DIA-based acquisition.

To specifically evaluate the reviewer's concern regarding the high number of unique peptide identifications in DDA-PASEF, we repeated the original five-method peptide-overlap analysis after replacing the original DDA-PASEF/FragPipe results with DDA-PASEF results processed by DIA-NN, while keeping DIA-, Slice-, Synchro- and midia-PASEF datasets processed with DIA-NN as in the main analysis. This analysis showed that DDA-PASEF still contributed the largest unique peptide set across all three gradients (Fig. R4 below; Supplementary Fig. 6b-d in the revised manuscript; lines 488-492), even when processed with DIA-NN. This indicates that the large DDA-specific peptide space is not primarily caused by FragPipe/MSFragger processing, but is strongly influenced by acquisition-mode characteristics. Several acquisition-level features may contribute to this observation. In our study, DDA-PASEF selected precursor ions from a broad m/z range during acquisition (from 100 to 1700), whereas the DIA-PASEF method used here covered a defined precursor m/z window range from 400 to 1000 using sequential isolation windows of 25 Da width.

As a result, DDA-PASEF can sample precursor ions outside or at the margins of the DIA isolation scheme, potentially generating peptide identifications not observed in the DIA-based datasets. In addition, the precursor-selective nature of DDA-PASEF can lead to run-to-run variation in which peptides are sampled, increasing the number of method-specific identifications when runs are combined. By contrast, DIA-based methods interrogate predefined isolation windows more consistently and therefore recover a more reproducible shared peptide core under the tested conditions.

We have revised the previous statements as (lines 146-150): "*Peptide-level overlap analysis revealed a large shared core among methods at all gradients, with overlap increasing at longer separations (Supplementary Fig. 1c–e). DDA-PASEF contributed the largest number of method-specific identifications, consistent with its precursor-selective acquisition strategy and broader precursor sampling space.*"

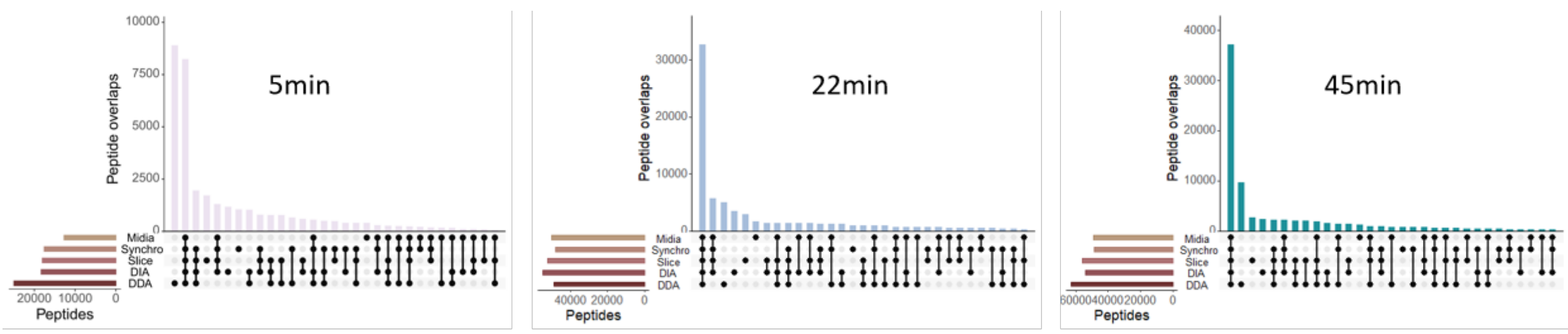

Fig. R4: Peptide identification overlaps between the five PASEF methods at 5-, 22- and 45-min gradients. DDA-PASEF data were processed in DIA-NN (version 2.3.2).

Comment 5: Since the authors have used Spectronaut 20 for their confirmatory analysis (Lines 145-146) , I wonder why that was not used as an algorithm of choice?

We thank the reviewer for this question. We used Spectronaut 20 as an independent confirmatory analysis rather than as the primary workflow, mainly because DIA-NN was better suited for the scale and design of this benchmark. DIA-NN is freely available for academic use, can be deployed on high-performance computing systems, and provided a unified, scalable workflow for processing DIA-, Slice-, Synchro- and midia-PASEF datasets. Spectronaut 20, while highly useful as an orthogonal validation tool, is commercial software requiring licensing (as a fact, we only obtained 1-month trial license at the time of analysis) and is less straightforward to integrate into large-scale automated cluster processing. Of note, during this review process, Thermo Scientific has announced DIA-NN as the main engine for DIA analysis in its commercial data analysis suite, demonstrating the acceptance and adoption of the underlying DIA-NN-based algorithms. We therefore retained DIA-NN as the primary analysis platform and used Spectronaut to confirm selected key trends.

Comment 6: Consider defining each PASEF mode in a short table for readers unfamiliar with Slice/Synchro/miDIA.

We thank the reviewer for this suggestion. Recent reviews provide broader summaries of PASEF variants (e.g., doi.org/10.1080/14789450.2024.2413092), and we agree that a short manuscript-specific table would be useful for interpreting the present study. Therefore, we have added a summary table to Supplementary Data 14 describing the general acquisition principle, precursor sampling strategy, and practical considerations for DDA-, DIA-, Slice-, Synchro-, and midia-PASEF (lines 638-641 in the revised manuscript).

Comment 7: Fig. 2F and Fig. 2G are excellent but dense. Consider adding small schematic icons to help readers interpret these figures.

We thank the reviewer for this suggestion. Rather than adding schematic icons that could further increase the visual complexity of these dense panels, we clarified the figure legend to better guide interpretation. We now explicitly state that Fig. 2f shows scaled peptide counts supporting taxonomic annotation, with darker red indicating higher peptide support and gray indicating species not detected in the corresponding replicates. We also clarified that Fig. 2g shows the distribution of microbial protein support per EC entry, where protein support refers to the number of microbial proteins annotated to each EC function.

We have revised the figure legends (lines 1001-1008 in the revised manuscript) in the manuscript as: *"**f** Scaled peptide counts supporting taxonomic annotation for species shared across the five PASEF methods in spike-in replicates (n = 9 per method). Each cell represents the relative number of peptides assigned to a given species (in columns) within each method (in rows), with darker red indicating higher peptide support. Gray indicates species not detected in the corresponding replicates. **g** Distribution of microbial protein support per EC entry across acquisition methods and gradients. Protein support refers to the number of microbial proteins annotated to each EC entry, providing a measure of functional annotation depth."*

Comment 8: Fig. 4C–D: The upward shift in Slice-PASEF intensities is important; consider quantifying the median shift.

We thank the reviewer for this helpful suggestion. To address this, we calculated paired abundance differences between Slice- and DIA-PASEF for features detected in both methods. Specifically, we computed $\Delta\log_2$ abundance as $\log_2(\text{Slice}) - \log_2(\text{DIA})$ separately for species-level abundances and species-specific peptide intensities in proximal and distal samples.

This analysis confirmed a systematic positive shift for Slice-PASEF at both levels (Fig. R5). At the species level, Slice-PASEF showed median abundance shifts of 2.51 in distal samples and 2.48 in proximal samples relative to DIA-PASEF. At the species-specific peptide level, the corresponding median shifts were 2.52 and 2.59, respectively.

We have added the median shifts generated here into the revised manuscript (lines 304-311) as: *"……Slice-PASEF exhibited a systematic upward shift in species abundance distributions (Fig. 4c; median Slice–DIA abundance shifts of 2.51 and 2.48 $\log_2$ units for distal and proximal samples, respectively). This shift was not driven by differences in the number of quantified species (Supplementary Fig. 4b) or species-specific peptides (Supplementary Fig. 4e). Instead, it reflected*

higher peptide-level intensities in Slice-PASEF (Fig. 4d; median Slice–DIA shifts of 2.52 and 2.59 log_2 units for distal and proximal samples, respectively)……"

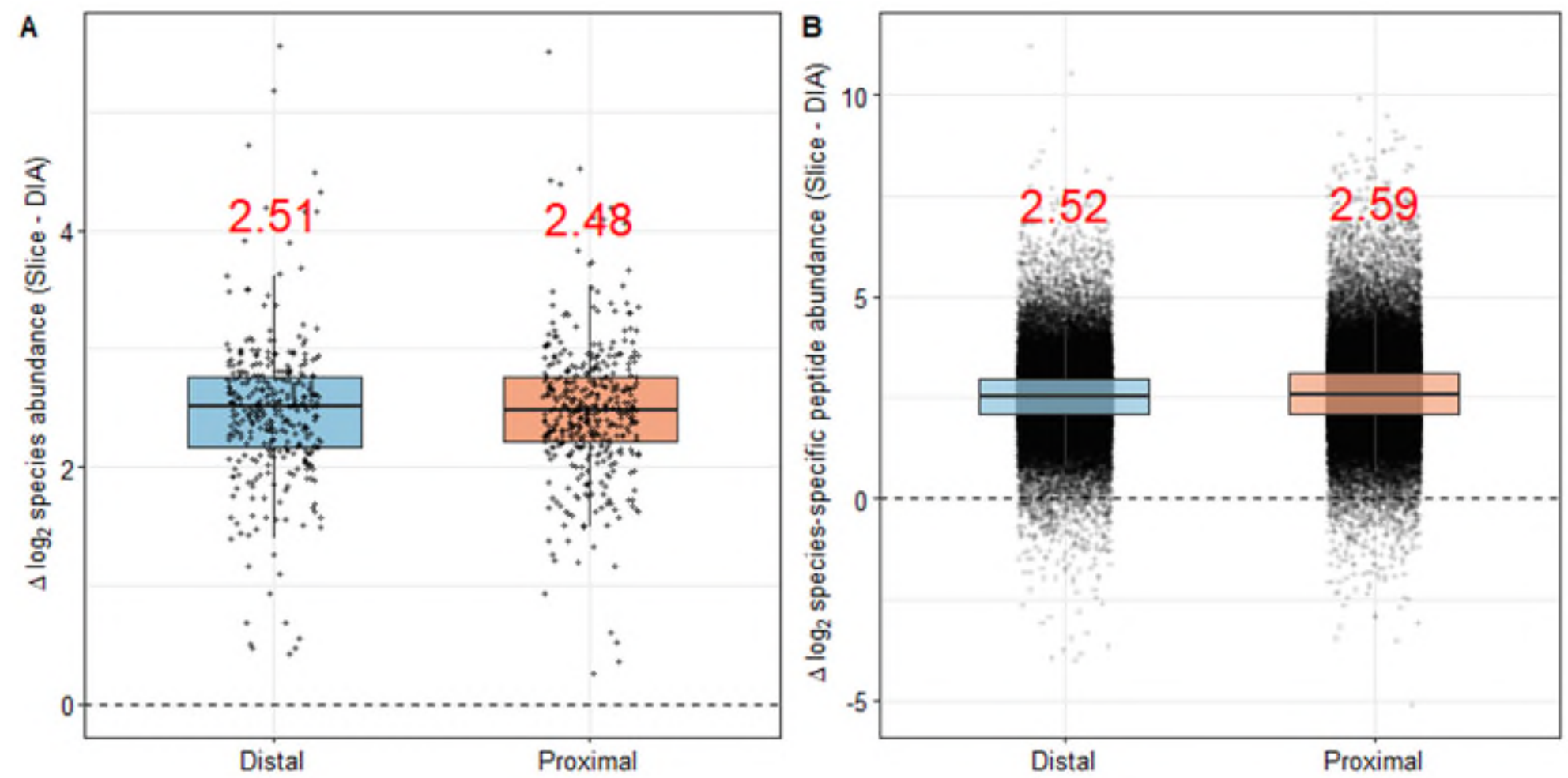

Fig. R5: Boxplots showing quantitative intensity shifts between Slice- and DIA-PASEF for species (A) and species-specific peptides (B). The red-label texts represent the median values of the corresponding distributions.

Comment 9: The microbial database construction section is detailed but could benefit from a schematic summarizing the multi-step database generation.

We thank the reviewer for this helpful suggestion. We agree that the microbial database construction workflow would benefit from a visual summary. We have therefore created a schematic workflow (Fig. R6) summarizing the microbial database generation and data processing steps for both the benchmark dataset and the murine validation dataset. This workflow has now been included as a new Supplementary Fig. 7 and referenced in the revised manuscript (lines 705-706; lines 751-752).

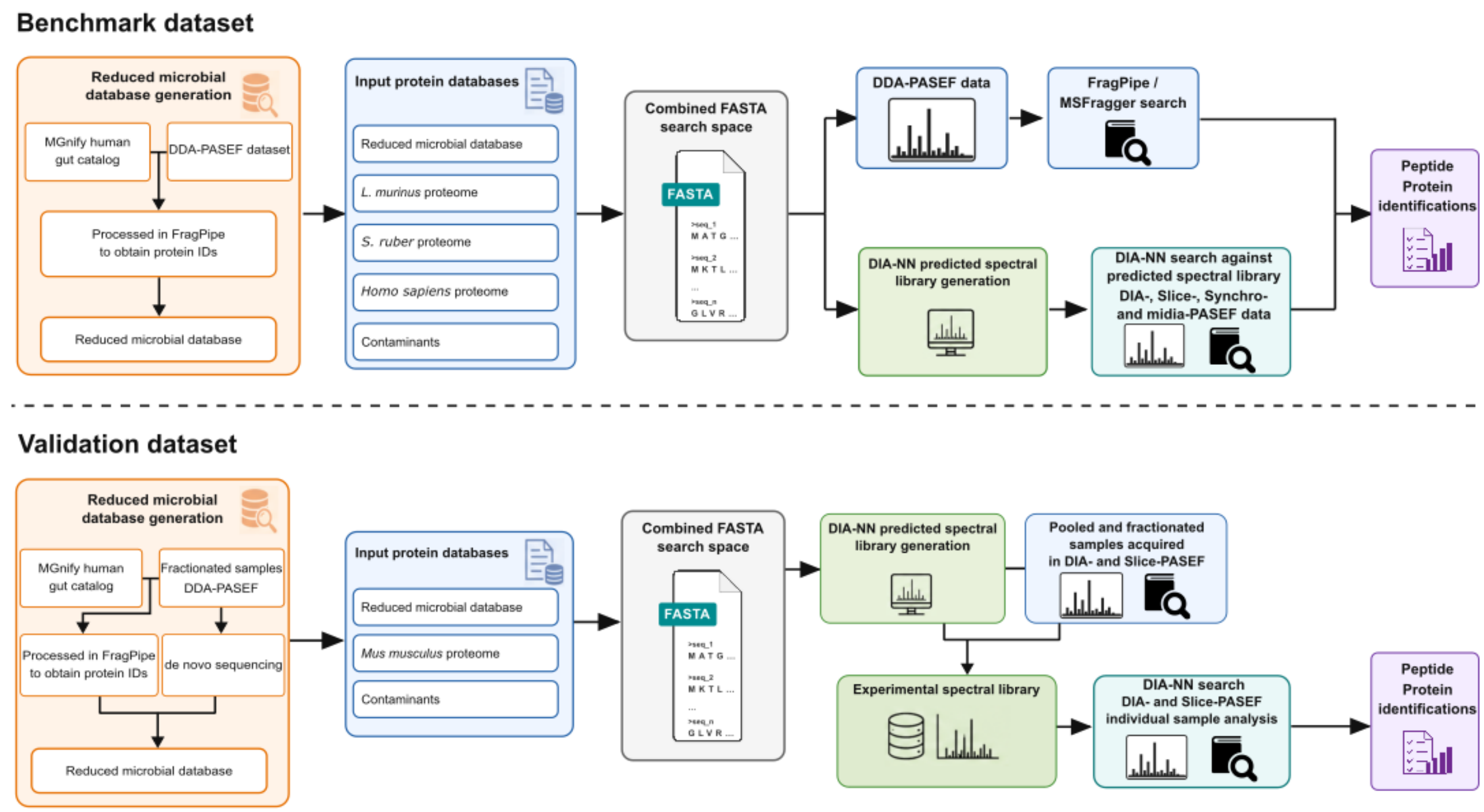

Fig. R6: Schematic workflows for microbial database construction and data processing strategies used in the study.

Comment 10: To maximize the utility of this work for the metaproteomics community, the authors should consider including a summary table which enlists the benefits of the five 'pentaPASEF' acquisition flavors for metaproteomics. This table can clearly delineate the specific benefits, trade-offs, and ideal use cases for each method based on the results of this study. This would provide a valuable decision-making roadmap for researchers selecting the most appropriate strategy for complex microbiome samples.

We thank the reviewer for this excellent suggestion. We agree that a concise decision-making summary would substantially improve the practical utility of the manuscript for the metaproteomics community. This point is closely related to the suggestion raised by Reviewer #1 (Comment 1) regarding the need to translate the benchmarking results into clearer practical recommendations.

In response, we have added a new summary figure (Fig. R1 above) in the revised manuscript (new Supplementary Fig. 3) that compares the five PASEF acquisition strategies across key analytical and practical dimensions, including identification depth, low-abundance sensitivity, quantitative precision and accuracy, taxonomic and functional profiling performance, and gradient-dependent behavior. We have also added a new Results/Discussion section to explicitly translate these findings into practical recommendations (lines 273-283 and lines 459-478 in the revised manuscript).

Comment 11: On Line 198, authors mention "few lower correlations observed for Slice-PASEF at 22 min (r < 0.9)" did you mean 45 min and not 22 mins?

We thank the reviewer for pointing this out and for giving us the opportunity to clarify the statement. We confirm that the text refers to Slice-PASEF at the 22-min gradient, not the 45-min gradient. In the 22-min Slice-PASEF dataset, the few lower Pearson correlations (r < 0.9) could be traced to one specific spike-in replicate (Spike-in:high, replicate 1), as stated in the manuscript. We also re-examined the Slice-PASEF 45-min dataset. Although some correlations were close to 0.9, these values were not attributable to a clear outlier pattern, as well as for few low correlations observed in midia-PASEF 45-min dataset.

We have revised the wording to make this clearer and avoid confusion as (lines 195-200): "*Intra-group Pearson correlations were high for most datasets and gradients (Supplementary Fig. 2a). The few lower correlations observed for Slice-PASEF at the 22-min gradient (r < 0.9) were attributable to one high spike-in replicate, whereas lower correlations observed in the 45-min Slice- and midia-PASEF datasets were not linked to a consistent outlier pattern (Supplementary Data 3).*"

Comment 12: A few typos appear ("sassword" on line 665 and Acknoledgements on line 770). Please correct.

We thank the reviewer for pointing out these typo errors. We have corrected these in the revised manuscript.

Reviewer #3 (Remarks to the Author):

The manuscript entitled "Systematic Evaluation of PASEF Acquisition Strategies in Complex Metaproteomes" presents a benchmarking study of different mass spectrometry data acquisition strategies for characterizing gut microbiome metaproteomes from identification, quantification, species-level, and functional perspectives.

General Comments:

Comment 1: The manuscript is well written, and the methods are generally sound. While some of the results were expected, if not already known (e.g., DDA vs. DIA), others are interesting, particularly the differences observed from the DIA-based methods. From an analytical standpoint, the benchmarking is fairly comprehensive. Nevertheless, I do have some comments regarding how some results are presented and discussed, as well as the lack of comprehensiveness from a computational perspective.

We thank the reviewer for the positive assessment of the manuscript, and we also appreciate the reviewer's recognition that the comparisons among DIA-based methods provide interesting insights beyond the expected DDA versus DIA differences. Regarding the suggestions and concerns raised by the reviewer, we provide point-by-point responses below.

Major Comments:

Comment 2: The fact that DDA and DIA workflows were not analyzed using the same metaproteome sequence database (database vs. library) is problematic. Why not use the library generated for the DIA-NN analysis to also perform the DDA analysis? This would reduce an additional source of variability that could contribute to the observed differences. This is somewhat akin to comparing apples and oranges.

We thank the reviewer for raising this important point, and we apologize for not describing the data search strategy clearly enough in the Methods section, which may have led to confusion. We agree that differences in database or library construction can introduce variability in metaproteomic benchmarking and should be explicitly described.

However, we would like to clarify that the DDA- and DIA-based analyses in our benchmark were not performed against different metaproteome sequence databases. The overview of database construction and data-processing steps is now summarized in new Supplementary Fig. 7 (see Fig. R6 above in response to Comment 9 from Reviewer #2). Specifically, DDA-PASEF files from the benchmark dataset were first searched against the MGnify human gut catalog using FragPipe/MSFragger, and a reduced microbial protein database was generated based on the identified proteins. This reduced microbial database was then combined with the reference proteomes of *L. murinus*, *S. ruber* and *Homo sapiens*, together with contaminant sequences. Importantly, this same combined FASTA search space was used both to process the DIA-based

datasets in DIA-NN and to re-process the DDA-PASEF dataset using FragPipe/MSFragger. Thus, the comparison was based on the same protein sequence database.

While not requested by the reviewer, it is fair to note that another source of variability that has not been tested previously (to the best of our knowledge) is the different signal processing implemented in FragPipe/MSFragger and DIA-NN. FragPipe/MSFragger performs a sequence database search by generating theoretical peptides and fragment masses from the FASTA file, whereas DIA-NN generates an in silico predicted spectral library from the same FASTA, including predicted fragment intensities, retention times, and ion mobility values. Following similar concerns raised by Reviewer #1, Comment 2 and Reviewer #2, Comment 4, we performed an additional reciprocal cross-validation analysis. Specifically, DIA-PASEF data were reprocessed using the FragPipe/diaTracer workflow, and DDA-PASEF data were reprocessed using DIA-NN v2.3.2, which recently introduced support for DDA data analysis. In both cases, the same protein sequence database was used. The results shown in Fig. R2 support that the major DDA versus DIA trends are not explained solely by data-analysis platform effects.

To clarify this point, we have revised the Methods section (lines 656–665) to explicitly state that the same combined FASTA search space was used for all benchmark datasets.

Comment 3: Another missing aspect of this paper is the impact of the spectral library itself. Metaproteome spectral library generation is an important area of research, and it has been shown that the choices made during library generation can substantially affect the final results. A comparison of different library sizes (large versus more focused libraries) would therefore be important.

We thank the reviewer for raising this important point. Indeed, several landmark studies have demonstrated the influence of spectral library size and sequence database construction in metaproteomic identification and quantification (doi.org/10.1371/journal.pone.0082981; doi.org/10.1128/msystems.00678-22; doi.org/10.1016/j.csbj.2022.01.018). In this study, however, our primary goal was to benchmark MS/MS acquisition strategies. To acknowledge the current know-how in the metaproteomics field and minimize database-dependent variation, the analysis of all MS/MS acquisition methods was processed using the same FASTA search space throughout the benchmark. Introducing multiple FASTA/spectral library sizes as a new variable would add major complexity to our study. A simple calculation of adding three different FASTA/spectral library sizes per method and gradient length would increase the data analysis necessary from the current 540 to 1620. A comprehensive evaluation of FASTA/spectral library sizes, database specificity, and their interaction with different PASEF acquisition modes would require a dedicated study alone.

To address this topic, we have added the following clarification to the revised Discussion (lines 540-547): "*Spectral library size and sequence database design are important determinants of metaproteomic performance. In this study, we used a consistent sequence database and a predicted-library strategy across acquisition modes to minimize database- and library-dependent*

variation and focus on acquisition-dependent differences. However, different database sizes and spectral library designs may influence identification depth, quantitative behavior, and false-discovery control, and should be systematically evaluated in future metaproteomic benchmarking studies."

Comment 4: Why require 3 taxa-specific peptides for taxon identification? A single taxa-specific peptide should be sufficient for inclusion in the analysis.

We thank the reviewer for raising this point. We agree that a single taxa-specific peptide can provide evidence for the presence of a taxon. However, in highly complex metaproteomes, taxonomic assignment based on a single peptide is more vulnerable to ambiguous or unstable assignments arising from sequence similarity, incomplete reference databases, peptide misidentification, or low-confidence method-specific detections. As our aim was to compare acquisition strategies rather than maximize the total number of taxa, we prioritized specificity and reproducibility over taxonomic depth (single-peptide sensitivity). This is particularly important in method benchmarking, where taxa supported by only one peptide could disproportionately inflate apparent method-specific differences.

In addition, this choice is consistent with established practice in our previous publications (e.g., doi.org/10.1038/s41467-025-61977-7; doi.org/10.3389/fmicb.2023.1258703), as well as with other studies, where three-peptide thresholds have been used (e.g., doi.org/10.1038/s41467-018-05357-4; doi.org/10.1080/19490976.2021.1994836) to balance taxonomic sensitivity and specificity. Moreover, recent benchmarking (doi.org/10.1016/j.mcpro.2024.100840) using artificial microbial communities showed that a three-peptide cutoff achieved substantially improved taxonomic accuracy compared with a one-peptide cutoff, including an increase in genus-level F-score from approximately 0.3 with one peptide to approximately 0.8 with three peptides (Figure 5C of the referenced study).

Comment 5: In Figure 3, it is unclear how the rank of a microbe is calculated. This should be clarified in the Methods.

We thank the reviewer for pointing this out. We agree that the calculation of microbial rank should be described more clearly in the manuscript.

In brief, species-level relative abundances were calculated from peptide intensities after taxonomic annotation. For each acquisition method and gradient, peptides assigned to the same species were first filtered to retain confidently annotated species supported by at least three taxon-specific peptides. Peptide intensities assigned to each species were then summed within each sample, and the resulting species-level intensity was divided by the total summed intensity of all retained species in that sample to obtain relative abundance. Species were ranked independently within each sample according to their relative abundance, with rank 1 corresponding to the most abundant species. For the rank-variability analysis (Fig. 3c), ranks were calculated per replicate sample, and the coefficient of variation of ranks across nine spike-in

replicates was used to assess compositional stability. For visualization of spike-in ranks (Fig. 3d), relative abundances were averaged across replicate injections for each method, gradient and spike-in level before ranking.

We have added the description to the Methods as "Calculation of relative species abundance rank" in the revised manuscript (lines 793-803) as: "*Species-level relative abundance ranks were calculated from taxonomically annotated peptide intensities. Species supported by at least three taxon-specific peptides were retained. For each sample, peptide intensities assigned to the same species were summed, and the species-level summed intensity was divided by the total summed intensity of all retained species to obtain relative abundance. Species were then ranked within each sample according to relative abundance, with rank 1 assigned to the most abundant species. For spike-in rank visualization, relative abundances were averaged across acquisition replicates for each method, gradient, and dilution level before ranking. For rank-variability analysis, ranks were calculated for each replicate sample, and the coefficient of variation of species ranks across spike-in replicates was used as a measure of compositional stability*."

Comment 6: In Figure 4B, why was the PCA analysis not performed directly on peptide or protein identifications or quantifications? In fact, this analysis should be performed for benchmarking shown in Figure 2. This would allow for a better visualization of the differences among acquisition strategies. Furthermore, the Figure 4B caption is not descriptive enough.

We thank the reviewer for this suggestion.

For Fig. 4b, our primary intention was to compare the biological microbial community profiles recovered by DIA- and Slice-PASEF in the murine colonic injury model, not raw peptide- or protein-level acquisition behavior. We therefore performed the PCA at the species-abundance level using Bray–Curtis dissimilarity, as this provides a biologically interpretable view of microbial community structure. Peptide- or protein-level profiles can be useful for technical benchmarking, but in the in vivo dataset it may be strongly influenced by different identification depths under experimental conditions, which were shown in Supplementary Fig. 3a. We have revised the Fig. 4b caption to explicitly state that the PCA was calculated from species-level relative abundance profiles using Bray–Curtis dissimilarity (lines 1029-1031): "***b**, Principal Coordinate Analysis based on Bray–Curtis dissimilarity of species-level relative abundance profiles quantified by DIA- and Slice-PASEF in proximal and distal colonic luminal samples across genotypes and time points."*

We also agree that a quantitative profile at peptide/protein-level is useful for the benchmarking dataset related to Fig. 2. In response, we added a new supplementary analysis visualizing acquisition-strategy differences based on peptide-level quantitative profiles across gradients (Fig. R7). This analysis provides an additional overview of how the five PASEF methods separate according to their peptide identification/quantification landscapes, and complements the identification counts and overlap analyses shown in Fig. 2 and Supplementary Fig. 1.

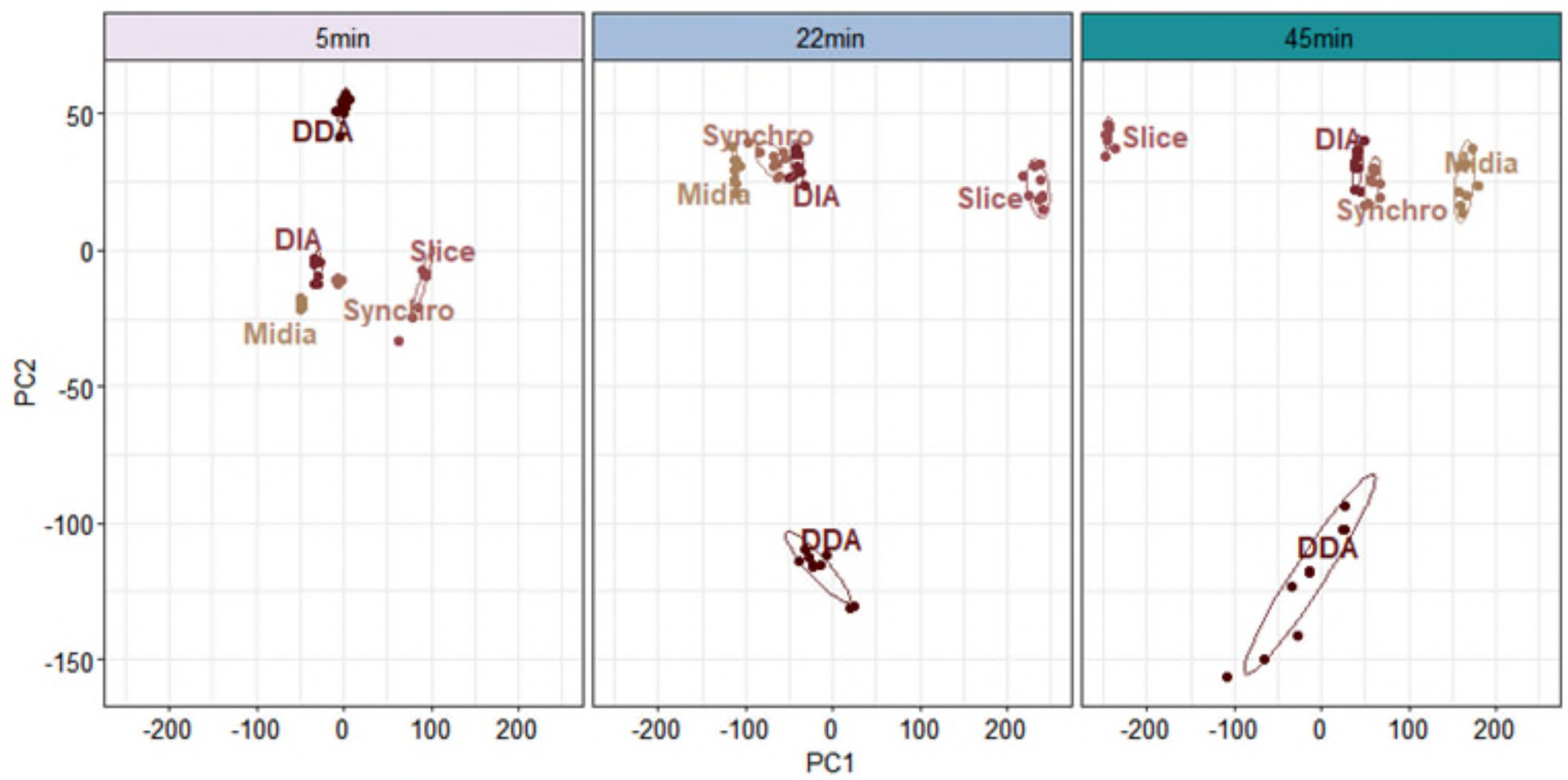

Fig. R7: Peptide-level quantitative profiles of five PASEF methods. Peptides detected in at least 80% of the replicates per gradient were retained, and the missing values were imputed with the minimum value.

We have added this analysis to Supplementary Fig. 1 and referenced it in the Results (lines 133-140) as: *"To further visualize global peptide-level differences among acquisition strategies, we performed PCA using peptide intensity profiles after averaging technical acquisition replicates within each gradient (Supplementary Fig. 1a). Samples clustered primarily by acquisition strategy, with DDA-PASEF separating most clearly from DIA-based methods and Slice-PASEF forming a distinct cluster among the DIA-derived approaches. This analysis indicates that PASEF modes differ not only in identification depth but also in the global peptide-level quantitative profiles they recover."*

Minor Comments:

Comment 7: Starting at line 156: "Nevertheless, DIA-based strategies retained higher recovery across gradients, demonstrating superior detection of low-abundance peptides in complex matrices." This statement is not true under all conditions.

We thank the reviewer for pointing this out, and we agree with the reviewer that the original wording was too broad. We have revised the sentence to more accurately reflect the data as follows (lines 155-157): *"Nevertheless, DIA-based strategies generally retained higher recovery across gradients and spike-in levels, indicating improved detection of low-abundance peptides in complex matrices."*

Comment 8: Figure 2F is acceptable, but showing the distribution of the number of peptides supporting each species, whether shared or unique, would be a much more effective way to present the data. To simplify the presentation, replicates could be pooled into one distribution.

We thank the reviewer for the suggestion. We agree that including species detected uniquely by individual methods can provide additional information on method-dependent taxonomic

annotation behavior. In total, 117 out 189 species were shared across the five acquisition methods, and these shared species were retained in Fig. 2f to enable direct comparison of peptide support across all methods and replicates.

We also evaluated the reviewer's suggestion by generating an all-species peptide-support matrix that included both shared and method-specific species (Fig. R8). This plot confirmed that the method-dependent peptide-support patterns observed in Fig. 2f were retained when uniquely detected species were included. In this all-species heatmap, many of the method-specific or non-shared species were associated with DIA- and Slice-PASEF (visible as a cluster in the mid-upper region of the heatmap), while a smaller cluster of species was mainly detected in DDA-PASEF (top-right of the heatmap). However, since method-specific/non-shared species introduce many missing entries and substantially reduce visual interpretability, we retained the shared-species heatmap in the main figure. To ensure transparency, we now provide the full peptide-support matrix for all species, including method-specific species, in the Source Data file accompanying Fig. 2f.

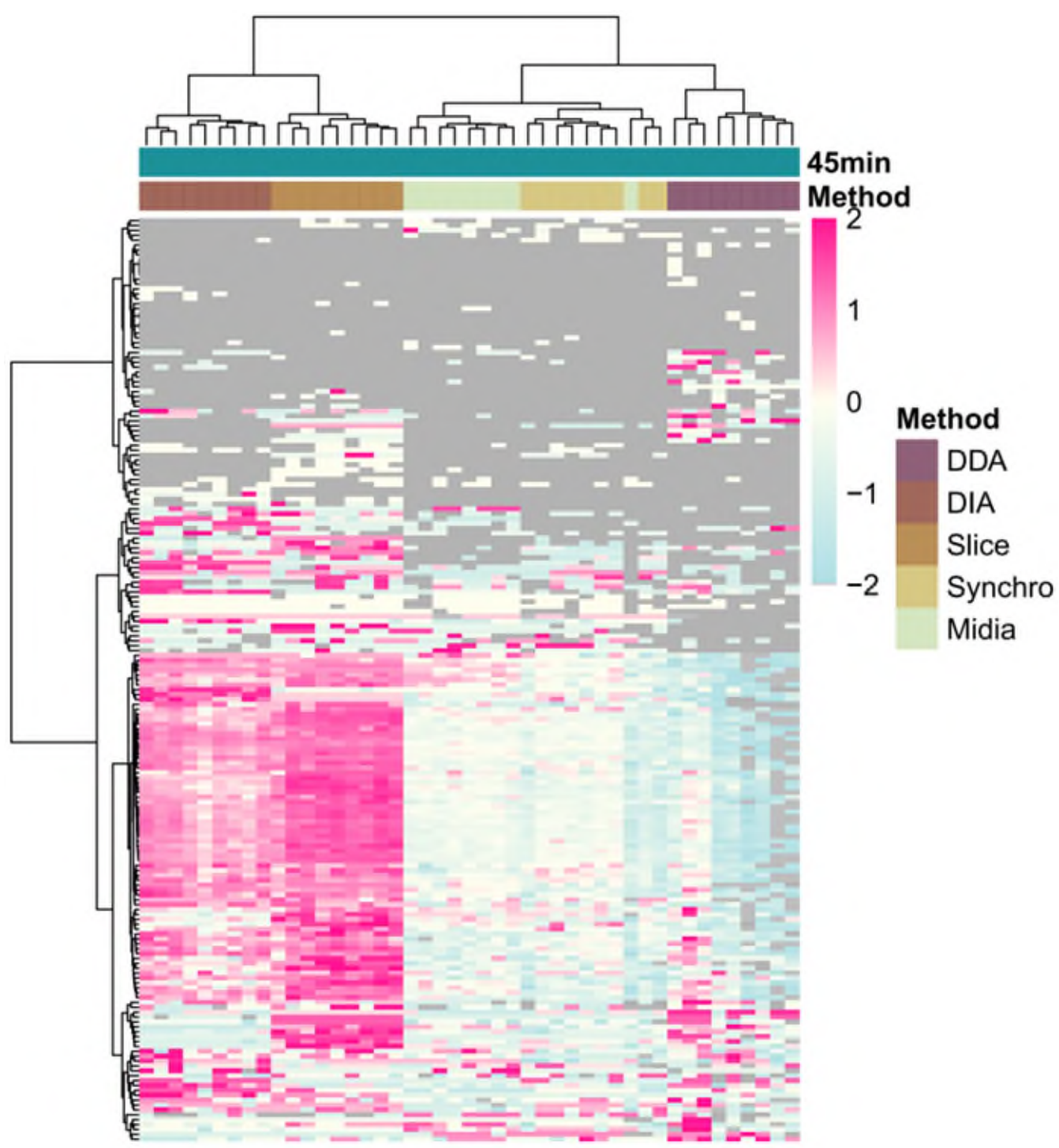

Fig. R8: Scaled peptide counts annotated for all species across five methods in spike-in replicates (n = 9 per method) at 45-min gradient. Gray color represents the species that were not detected in the respective replicate.

We also considered the reviewer's suggestion to pool replicates into a single distribution. However, in Fig. 2f our goal was to visualize not only the number of peptides supporting each species assignment, but also the consistency of this support across acquisition methods and spike-in replicate groups. Pooling all replicates would simplify the presentation, but it would remove information on replicate-level reproducibility, which is central to our comparison of acquisition

strategies. We therefore retained the original heatmap format, which preserves replicate-level information while providing the full peptide-support matrix for all detected species in the Source Data file.

Comment 9: At line 202, the authors state: "DDA-PASEF displayed a pronounced gradient dependence, with variability decreasing at longer separations, whereas DIA-based methods were less affected by gradient length." The average CVs should be provided in the text.

We thank the reviewer for this helpful suggestion. We have now added the average replicate-level median CVs for DDA-PASEF directly in the text to support the statement on gradient-dependent variability (lines 202-208): *"Interestingly, DDA-PASEF displayed a pronounced gradient dependence, with variability decreasing at longer separations (average replicate-level median CV: 15.2% at 5 min, 11.5% at 22 min, and 9.64% at 45 min). In contrast, DIA-based methods showed less uniform gradient dependence; for example, Slice-PASEF maintained comparatively low median CVs at 5 and 22 min but showed higher variability at 45 min (average replicate-level median CV: 7.85%, 9.53% and 13.5%, respectively)."*

Comment 10: At line 320, the term "borderline statistical behavior" is overly optimistic. Results that fail multiple hypothesis correction are simply not statistically significant.

We thank the reviewer for this comment. We have revised the text to avoid implying statistical significance for features that did not pass multiple-testing correction (lines 338-343): *"Inspection of raw P values revealed that many method-unique species showed nominal differences in the alternative method but did not pass multiple-testing correction (Supplementary Fig. 4g; Supplementary Data 10). These features were therefore not statistically significant in the alternative method, suggesting that method uniqueness often reflected differences in statistical power or detection sensitivity rather than discordant biology."*
