# Supplementary material for: Systematic evaluation of PASEF acquisition strategies in complex metaproteomes
Source: Nat Commun. 2026 Aug 3;17:7406. doi: 10.1038/s41467-026-75845-5 (PMC13434454; doi:10.1038/s41467-026-75845-5)
Supplement: Supplementary file 3 [file 41467_2026_75845_MOESM1_ESM.pdf]

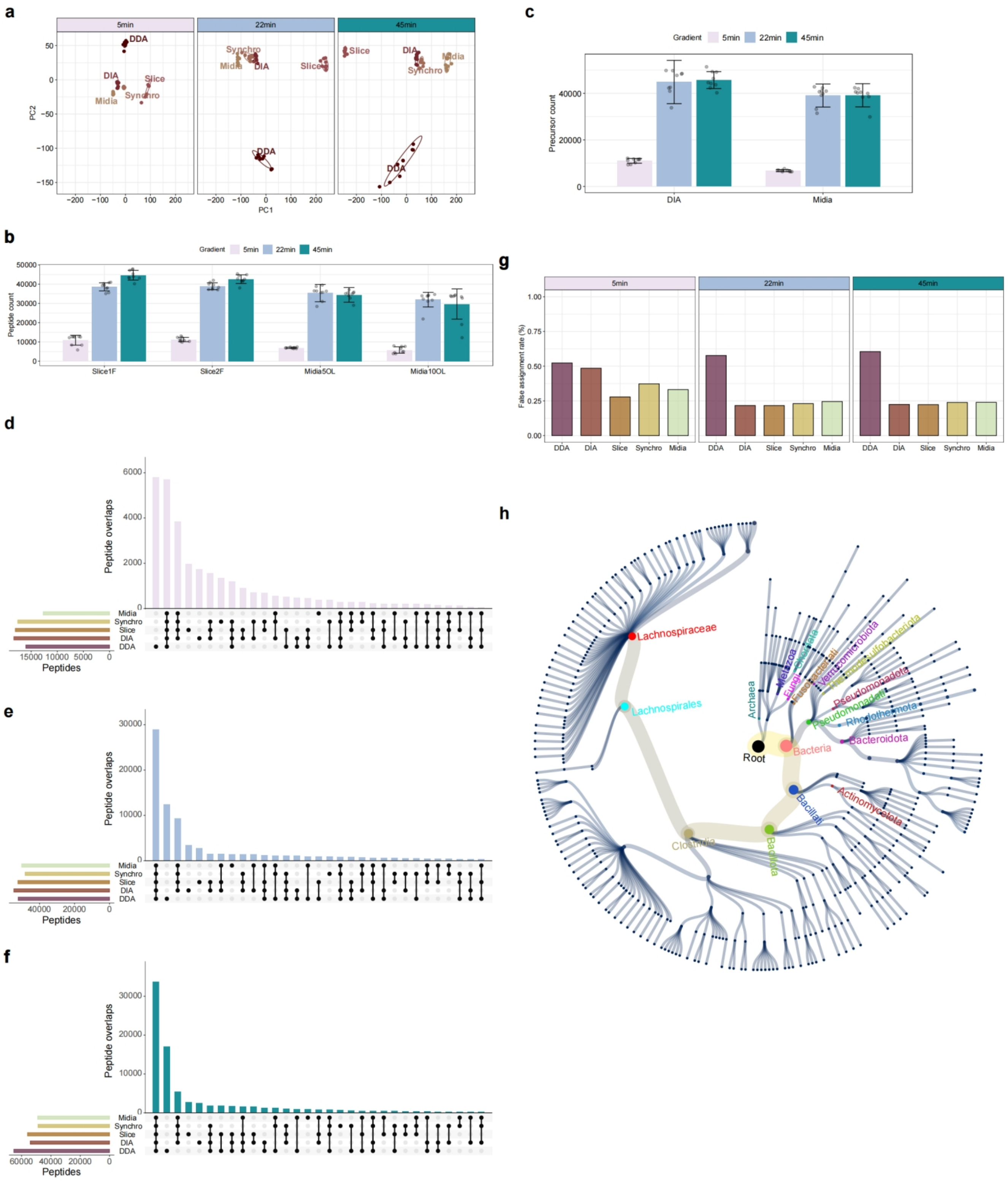


**Supplementary Fig. 1** PentaPASEF methods evaluation. **a** Peptide-level quantitative profiles of five PASEF methods. Peptides detected in at least 80% of the replicates per gradient were retained, and the missing values were imputed with the minimum value. **b** Comparison of identified peptide numbers obtained using alternative Slice-PASEF frame settings and midia-PASEF window overlap schemes across LC gradients. The bars represent the means of spike-in replicates (n = 9), and the error bars show the standard deviations. **c** Identified precursors in DIA- and midia-PASEF datasets analyzed with Spectronaut 20. The bars represent the means of spike-in replicates (n = 9), and the error bars show the standard deviations. **d–f** Peptide identification overlaps between the five PASEF methods at 5- (**d**), 22- (**e**) and 45-min (**f**) gradients. **g** Frequency of falsely assigned heavy-labeled *L. murinus* precursors and *S. ruber* precursors in non-spike controls across acquisition methods and gradients. **h** Example (based on the annotation of samples from

DIA-PASEF with 22-min gradient) of the taxonomic composition of the fecal background sample. Plot was modified based on MetaTree[1]. Source data for Supplementary Fig. 1a-g are provided in the Source Data file.

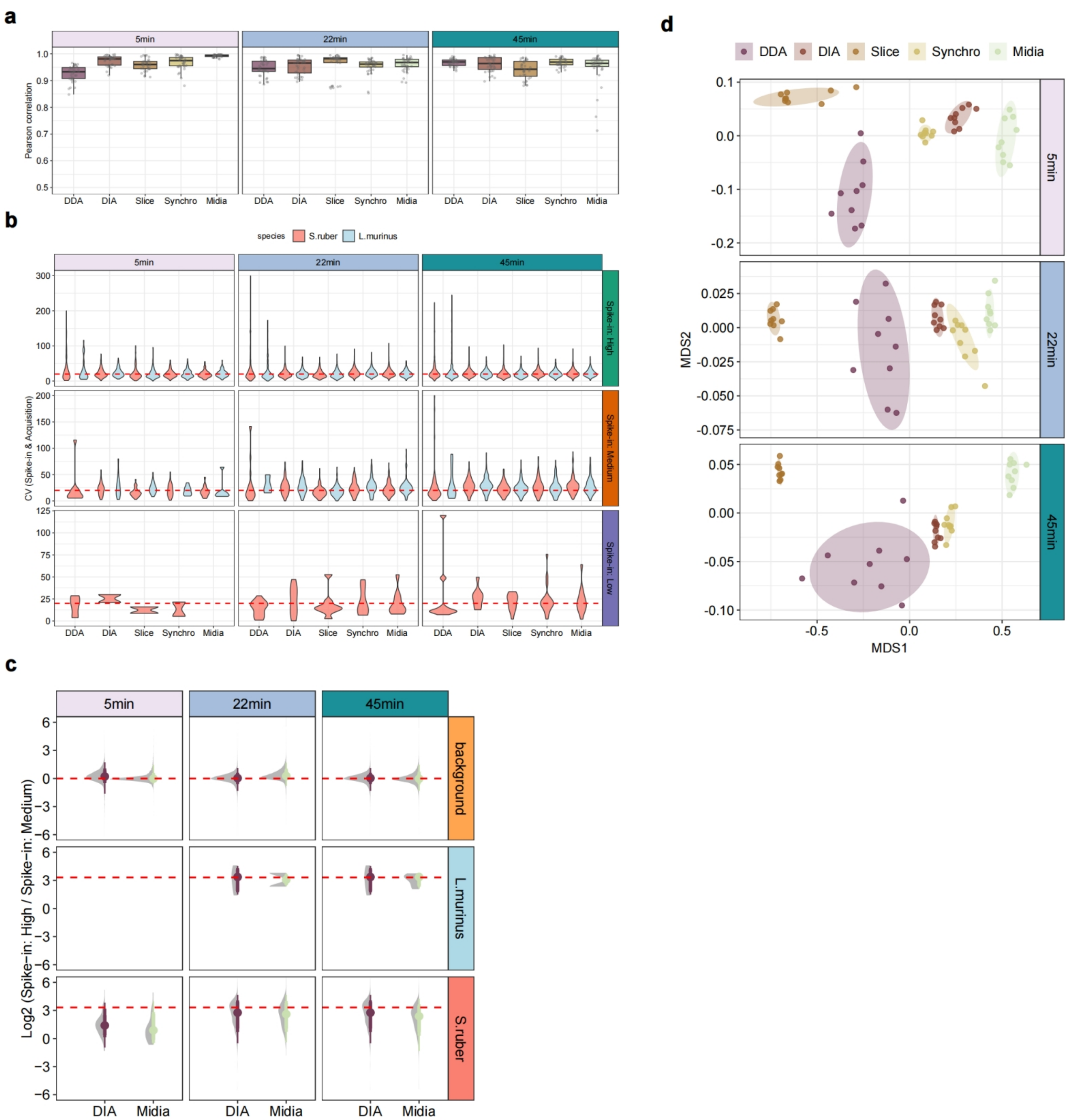


**Supplementary Fig. 2** Reproducibility and diversity analyses supporting quantitative evaluation of PASEF methods. **a** Intra-group Pearson correlation matrices of peptide intensities across spike-in replicates (n = 9) for each method and gradient. Each box represents the interquartile range, spanning from the 25th percentile (lower bound of the box) to the 75th percentile (upper bound). The center line indicates the median (50th percentile). **b** CV (coefficient of variation) distributions of quantified peptides for *L. murinus* and *S. ruber* at different spike-in levels and gradients. **c** Evaluation of quantitative accuracy using Spectronaut 20 quantification for DIA- and midia-PASEF datasets. **d** Bray–Curtis beta diversity analysis of species abundance profiles across spike-in replicates (n = 9) and PASEF methods. Source data for Supplementary Fig. 2 are provided in the Source Data file.

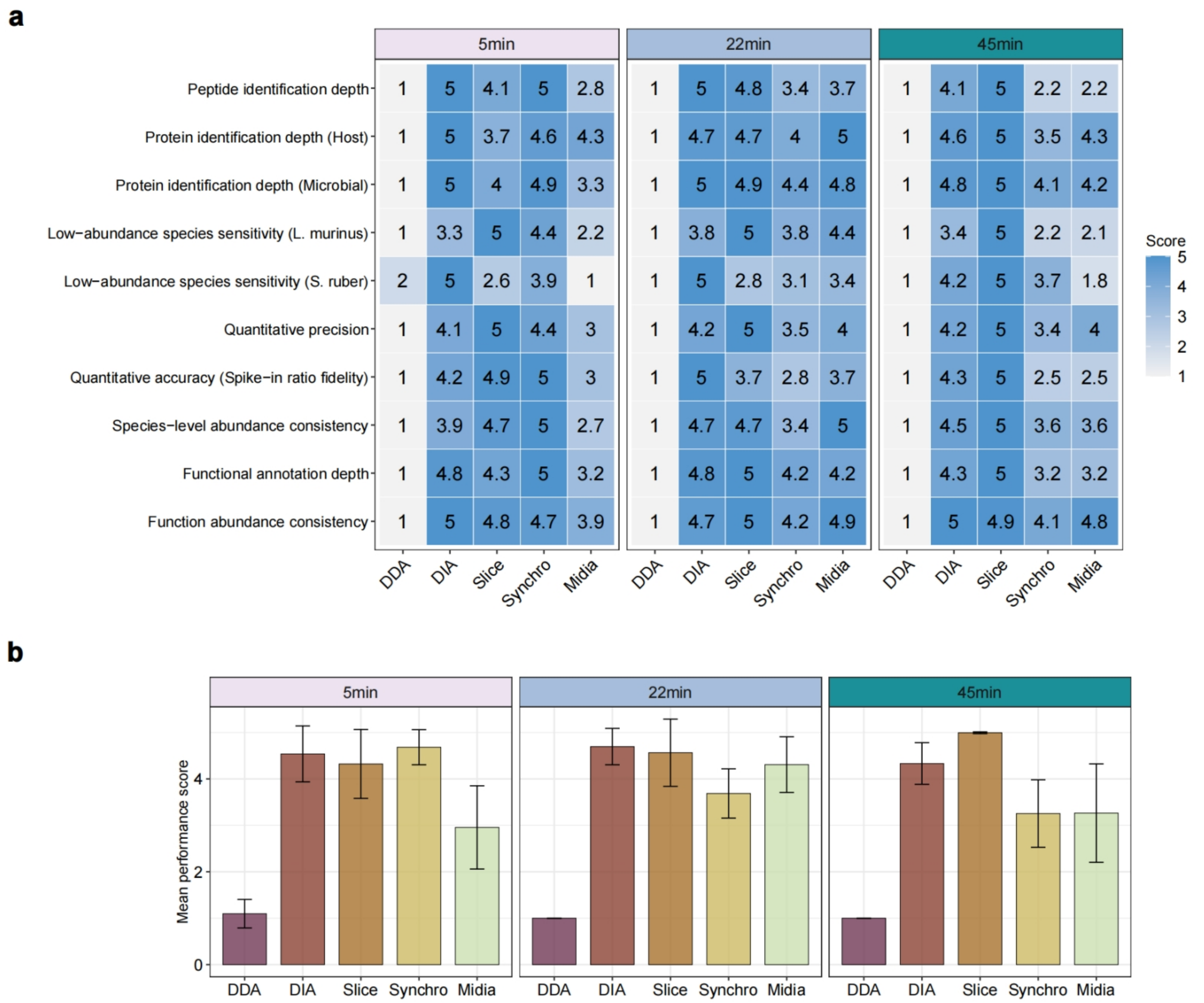


**Supplementary Fig. 3** Data-derived practical guidance for PASEF method selection. **a** Normalized performance scores for five PASEF acquisition strategies across gradients and analytical evaluation criteria. Scores range from 1 to 5, with higher values indicating better performance. **b** Mean performance score across evaluation criteria for each acquisition strategy and gradient. Bars indicate mean ± SD across the scored criteria. Source data for Supplementary Fig. 3 are provided in the Source Data file.

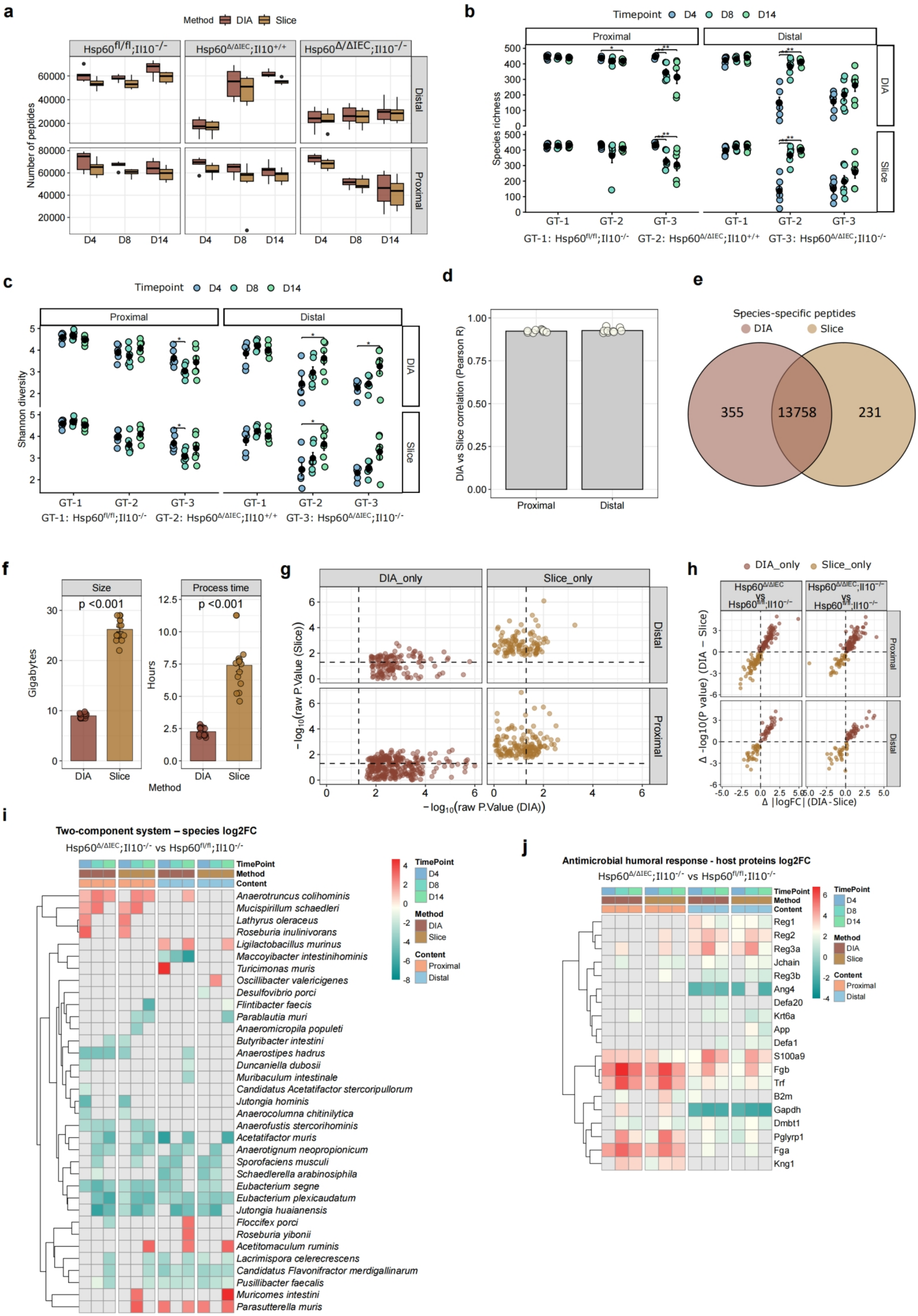

a
Method DIA Slice
Hsp60fl/fl;Il10-/-
Hsp60Δ/ΔIEC;Il10+/+
Hsp60Δ/ΔIEC;Il10-/-
Distal
Proximal
Number of peptides
D4 D8 D14
b
Timepoint D4 D8 D14
Proximal
Distal
DIA
Slice
Species richness
GT-1 GT-2 GT-3
GT-1: Hsp60fl/fl;Il10-/- GT-2: Hsp60Δ/ΔIEC;Il10+/+ GT-3: Hsp60Δ/ΔIEC;Il10-/-
c
Timepoint D4 D8 D14
Proximal
Distal
DIA
Slice
Shannon diversity
GT-1 GT-2 GT-3
GT-1: Hsp60fl/fl;Il10-/- GT-2: Hsp60Δ/ΔIEC;Il10+/+ GT-3: Hsp60Δ/ΔIEC;Il10-/-
d
DIA vs Slice correlation (Pearson R)
Proximal
Distal
e
Species-specific peptides
DIA Slice
355
13758
231
f
Size
Process time
p <0.001
Gigabytes
Hours
DIA Slice
Method
g
DIA_only
Slice_only
Distal
Proximal
- log10(raw P.Value (Slice))
- log10(raw P.Value (DIA))
h
DIA_only Slice_only
Hsp60Δ/ΔIEC vs Hsp60fl/fl;Il10-/-
Hsp60Δ/ΔIEC;Il10-/- vs Hsp60fl/fl;Il10-/-
Proximal
Distal
Δ -log10(P value) (DIA − Slice)
Δ |logFC| (DIA - Slice)
i
Two-component system – species log2FC
Hsp60Δ/ΔIEC;Il10-/- vs Hsp60fl/fl;Il10-/-
TimePoint
Method
Content
D4
D8
D14
DIA
Slice
Proximal
Distal
Anaerotruncus colihominis
Mucispirillum schaedleri
Lathyrus oleraceus
Roseburia inulinivorans
Ligilactobacillus murinus
Maccoyibacter intestinihominis
Turicimonas muris
Oscillibacter valericigenes
Desulfovibrio porci
Flintibacter faecis
Parablautia muri
Anaeromicropila populeti
Butyribacter intestini
Anaerostipes hadrus
Duncaniella dubosii
Muribaculum intestinale
Candidatus Acetatifactor stercoripullorum
Jutongia hominis
Anaerocolumna chitinilytica
Anaerofustis stercorihominis
Acetatifactor muris
Anaerotignum neopropionicum
Sporofaciens musculi
Schaedlerella arabinosiphila
Eubacterium segne
Eubacterium plexicaudatum
Jutongia huaianensis
Floccifex porci
Roseburia yibonii
Acetitomaculum ruminis
Lacrimispora celerecrescens
Candidatus Flavonifractor merdigallinarum
Pusillibacter faecalis
Muricomes intestini
Parasutterella muris
j
Antimicrobial humoral response - host proteins log2FC
Hsp60Δ/ΔIEC;Il10-/- vs Hsp60fl/fl;Il10-/-
TimePoint
Method
Content
Reg1
Reg2
Reg3a
Jchain
Reg3b
Ang4
Defa20
Krt6a
App
Defa1
S100a9
Fgb
Trf
B2m
Gapdh
Dmbt1
Pglyrp1
Fga
Kng1

**Supplementary Fig. 4** Comparison of DIA- and Slice-PASEF in the murine colonic dataset. **a** Number of quantified peptides across DIA- and Slice-PASEF in proximal and distal colon samples at each timepoint. Each box (n = 6 per box) represents the interquartile range, spanning from the 25th percentile (lower bound of the box) to the 75th percentile (upper bound). The center line indicates the median (50th percentile). **b** Number of confidently detected species in all sample conditions. Asterisks indicate statistical significance from a two-sided Wilcoxon rank-sum test for comparisons: * represents $p < 0.05$, ** represents $p < 0.01$. **c** Shannon diversity indices across genotypes, time points, and colonic regions for DIA- and Slice-PASEF. Asterisks indicate statistical significance from a two-sided Wilcoxon rank-sum test for comparisons: * representing $p < 0.05$. **d** Pearson's correlation of species-level abundances between DIA- and Slice-PASEF for proximal and distal samples. **e** Venn diagram showing the overlap of species-specific peptides identified by DIA- and Slice-PASEF. **f** Comparison of raw file sizes (Gigabytes) and DIA-NN processing times (hours) between DIA- and Slice-PASEF acquisitions. **g** Comparison of raw P values (−log10) obtained from differential abundance testing for significantly regulated method-dependent species in proximal and distal samples. **h** Difference in log2 fold change (log2FC) and statistical significance (−log10 P value) for significantly regulated method-dependent species in both methods across contrasts. **i** Heatmap of $\log_2$ fold changes for species associated with the KEGG "Two-component system" pathway across time points, methods, and colonic sites in the contrast of Hsp60$^{\Delta/\Delta IEC}$;Il10$^{-/-}$ vs Hsp60$^{fl/fl}$;Il10$^{-/-}$. **j** Heatmap of $\log_2$ fold changes for selected host proteins associated with antimicrobial humoral response functions across time points and colonic sites in the contrast of Hsp60$^{\Delta/\Delta IEC}$;Il10$^{-/-}$ vs Hsp60$^{fl/fl}$;Il10$^{-/-}$. Source data for Supplementary Fig. 4a-d and 4g-j are provided in the Source Data file.

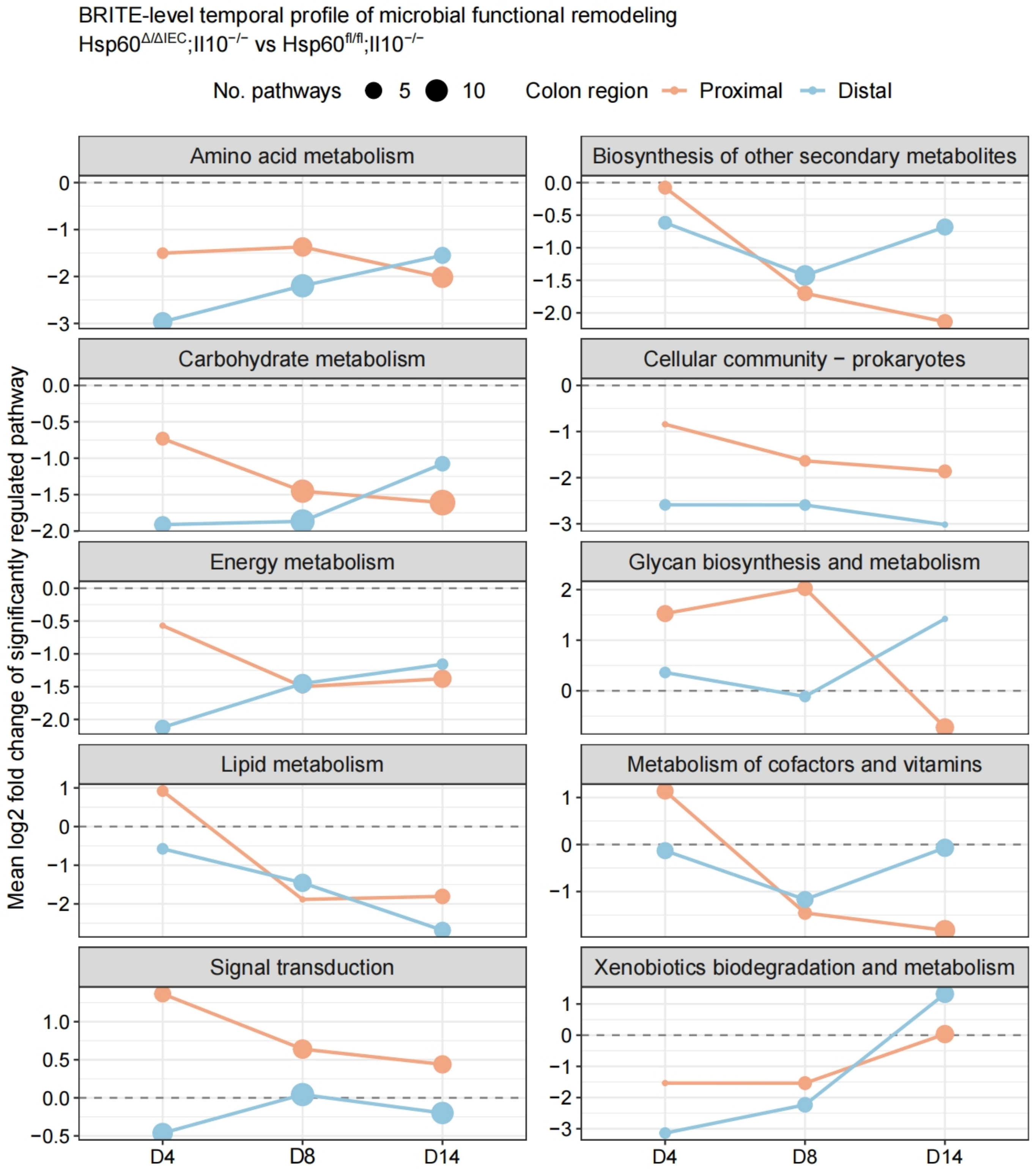


**Supplementary Fig. 5** BRITE-level temporal profiles of microbial functional remodeling. Mean $\log_2$ fold changes of significantly regulated microbial KEGG pathways are shown for $Hsp60^{\Delta/\Delta IEC};Il10^{-/-}$ mice compared with $Hsp60^{fl/fl};Il10^{-/-}$ controls across D4, D8 and D14. Pathways were grouped by KEGG BRITE functional categories. Point size represents the number of significantly regulated pathways within each BRITE category at the corresponding time point. Source data for Supplementary Fig. 5 are provided in the Source Data file.

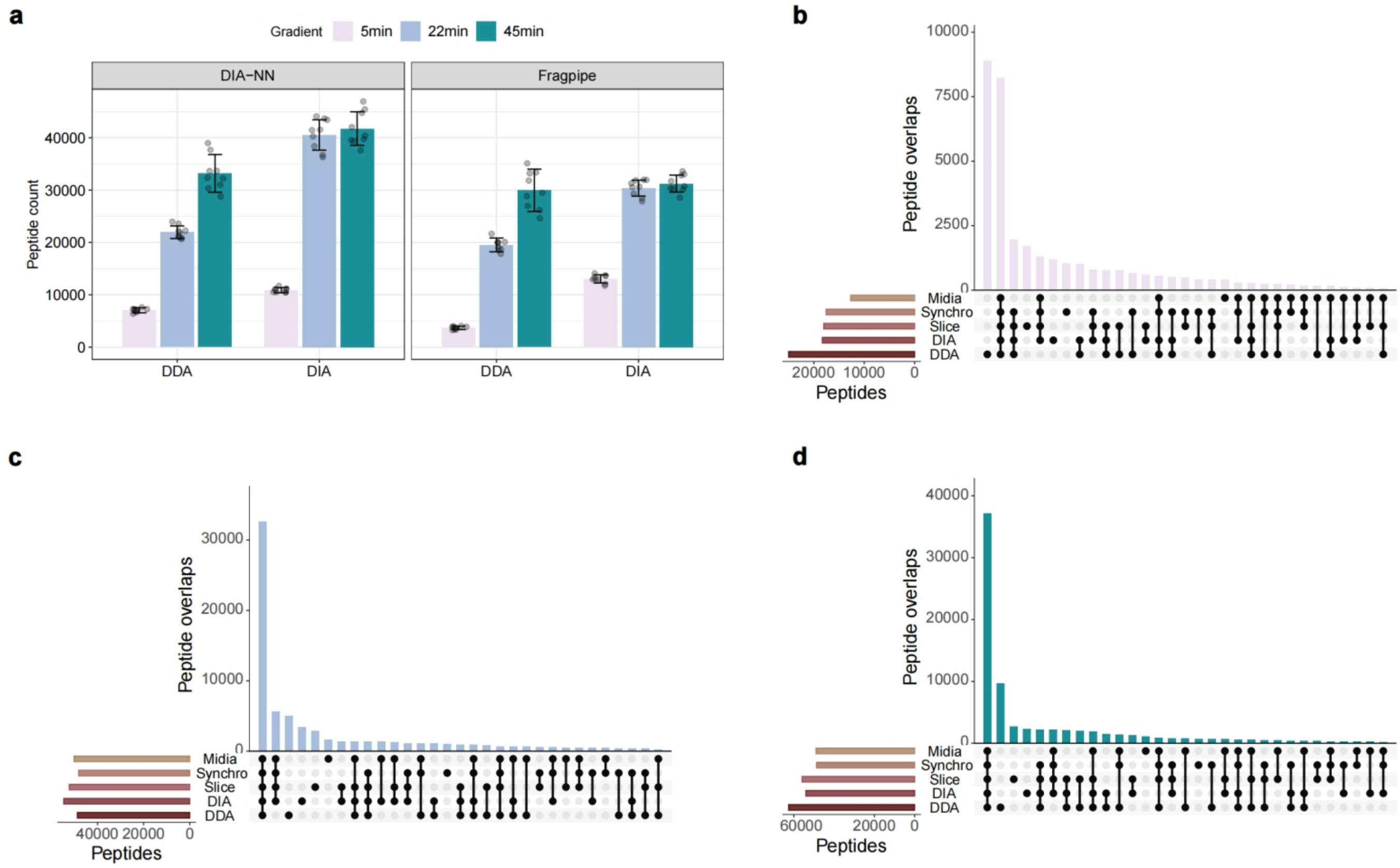


**Supplementary Fig. 6** Cross-platform validation of DDA- and DIA-PASEF peptide identifications. **a** Peptide identifications obtained from DDA- and DIA-PASEF data processed using DIA-NN or FragPipe/MSFragger-based workflows across 5-, 22- and 45-min gradients. Bars show mean peptide counts, with points representing individual replicate measurements (n = 9). **b–d** Five-method peptide-overlap analysis after replacing the original DDA-PASEF/FragPipe results with DDA-PASEF data processed by DIA-NN, while DIA-, Slice-, Synchro- and midia-PASEF were retained from the DIA-NN workflow. UpSet plots show peptide overlaps across the five acquisition strategies at 5 min (**b**), 22 min (**c**) and 45 min (**d**). Source data for Supplementary Fig. 6 are provided in the Source Data file.

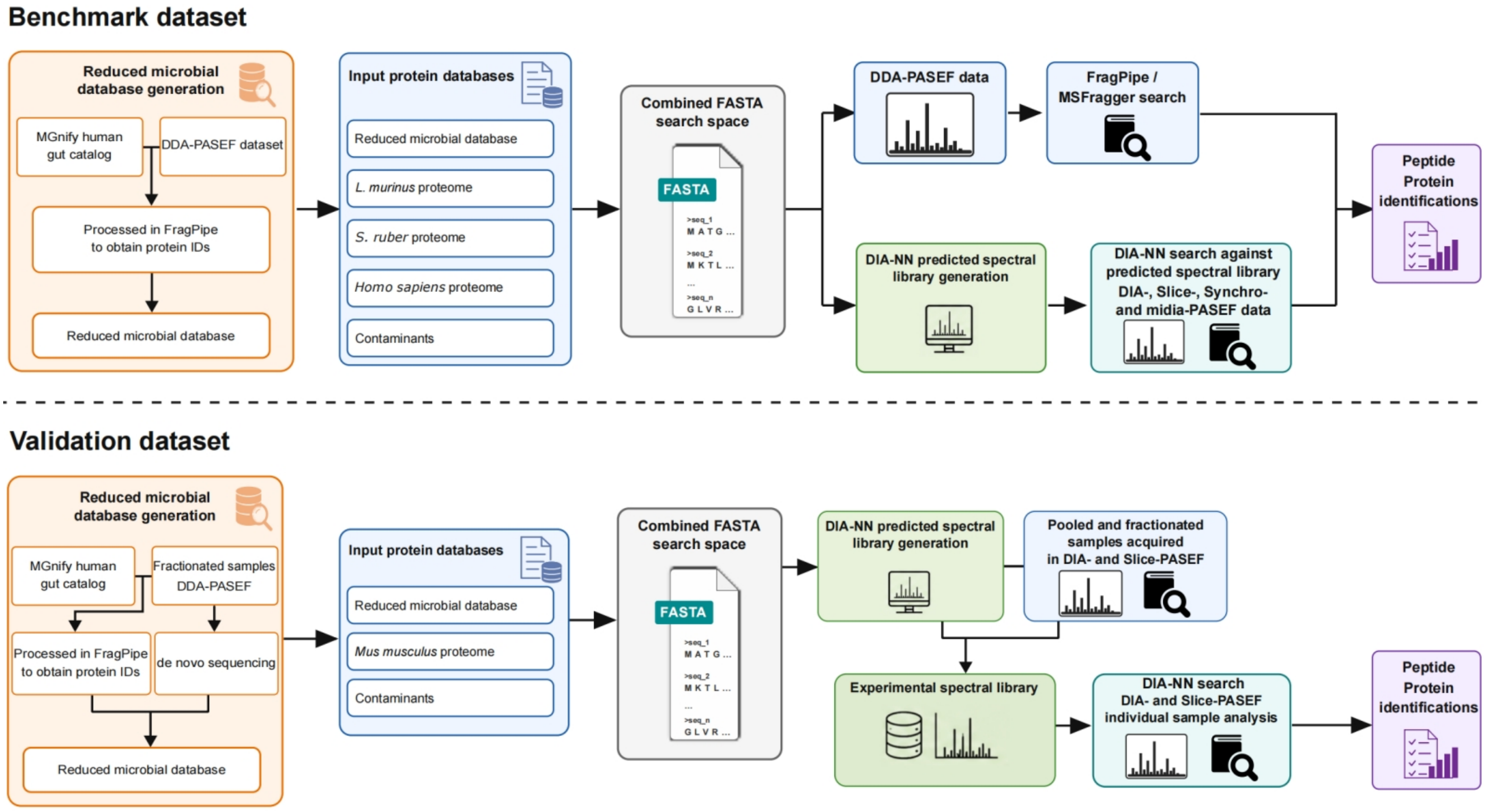


**Supplementary Fig. 7** Schematic workflows for microbial database construction and data processing strategies used in the study.

## References


1. Wu Q, Zhang A, Ning Z, Figeys D. MetaTree: an interactive web platform for aligned hierarchical data visualization and multi-group comparison. *BMC Bioinformatics*,  (2026).
